# The Fe(II)-oxidizing *Zetaproteobacteria*: historical, ecological and genomic perspectives

**DOI:** 10.1093/femsec/fiz015

**Published:** 2019-01-30

**Authors:** Sean M McAllister, Ryan M Moore, Amy Gartman, George W Luther, David Emerson, Clara S Chan

**Affiliations:** 1 School of Marine Science and Policy, University of Delaware, 700 Pilottown Road, 204 Cannon Lab, Lewes, Delaware, USA 19958; 2 Center for Bioinformatics and Computational Biology, University of Delaware, 15 Innovation Way, 205 Delaware Biotechnology Institute, Newark, Delaware, USA 19711; 3 Bigelow Laboratory for Ocean Sciences, 60 Bigelow Drive, East Boothbay, Maine, USA 04544; 4 Department of Geological Sciences, University of Delaware, 101 Penny Hall, Newark, Delaware, USA 19716

**Keywords:** *Zetaproteobacteria*, Fe(II) oxidation, microbial ecology, hydrothermal vents, marine Fe cycling, phylogenetic analysis

## Abstract

The *Zetaproteobacteria* are a class of bacteria typically associated with marine Fe(II)-oxidizing environments. First discovered in the hydrothermal vents at Loihi Seamount, Hawaii, they have become model organisms for marine microbial Fe(II) oxidation. In addition to deep sea and shallow hydrothermal vents, *Zetaproteobacteria* are found in coastal sediments, other marine subsurface environments, steel corrosion biofilms and saline terrestrial springs. Isolates from a range of environments all grow by autotrophic Fe(II) oxidation. Their success lies partly in their microaerophily, which enables them to compete with abiotic Fe(II) oxidation at Fe(II)-rich oxic/anoxic transition zones. To determine the known diversity of the *Zetaproteobacteria*, we have used 16S rRNA gene sequences to define 59 operational taxonomic units (OTUs), at 97% similarity. While some *Zetaproteobacteria* taxa appear to be cosmopolitan, others are enriched by specific habitats. OTU networks show that certain *Zetaproteobacteria* co-exist, sharing compatible niches. These niches may correspond with adaptations to O_2_, H_2_ and nitrate availability, based on genomic analyses of metabolic potential. Also, a putative Fe(II) oxidation gene has been found in diverse *Zetaproteobacteria* taxa, suggesting that the *Zetaproteobacteria* evolved as Fe(II) oxidation specialists. In all, studies suggest that *Zetaproteobacteria* are widespread, and therefore may have a broad influence on marine and saline terrestrial Fe cycling.

## Teaser

Review and synthesis of the discovery, ecology, and genetic potential of the most prolific marine Fe-oxidizing clade, the Zetaproteobacteria.

## INTRODUCTION

Fe in marine environments is a study in contrasts. It is often a limiting nutrient in the open ocean, while the basaltic ocean crust and many sediments have abundant Fe. This stark difference is due to the redox chemistry of Fe, which is present as Fe(II) in basalt and anoxic groundwater, but rapidly oxidizes to Fe(III) in oxic ocean water, precipitating as Fe(III) minerals. This oxidation was assumed to be dominated by rapid abiotic oxidation at circumneutral pH, but the discovery of the Fe(II)-oxidizing *Zetaproteobacteria* in marine environments gave proof that the process can be driven by microbes. First proposed as a class in 2007 (Emerson *et al*. 2007), *Zetaproteobacteria* have since been widely observed in deep sea and coastal environments. All isolates are obligate autotrophs and can couple Fe(II) oxidation to oxygen respiration, producing highly reactive Fe(III) oxyhydroxides that can adsorb or coprecipitate nutrients and metals (e.g. Laufer et al. 2017). However, despite the biogeochemical importance of microbial Fe(II) oxidation, we are just beginning to learn about Fe(II)-oxidizer distribution and how they function and influence marine ecosystems. With a recent surge of culturing and sequencing, there is now a substantial set of data from which we can glean broader insights into microbial Fe(II) oxidation in marine and other saline habitats.

The goal of this paper is to review our current knowledge of marine Fe(II)-oxidizers through the lens of this increasingly well-established class of Fe(II)-oxidizing bacteria (FeOB). We begin by describing the discovery of this novel class at an Fe(II)-rich hydrothermal system at Loihi Seamount (also spelled Lō’ihi Seamount) in Hawaii. We lay out the evidence for microbially-driven Fe(II) oxidation in this marine system, including new kinetics results from experiments with the Loihi isolate and model *Zetaproteobacteria, Mariprofundus ferrooxydans* PV-1. Work at Loihi has inspired numerous studies of *Zetaproteobacteria* isolates, biominerals and environmental distribution. In addition to reviewing these, we present a comprehensive reanalysis of *Zetaproteobacteria* diversity and distribution, enabled by the newly developed ZetaHunter classification program (McAllister, Moore and Chan 2018), to gain insights into Zetaproteobacteria niches (sets of conditions favorable for growth). We then use current genomic evidence to evaluate whether all members of this class have the potential to oxidize Fe(II) and further describe *Zetaproteobacteria* niches based on inferred metabolic potential. Finally, we discuss our perspectives on open questions in *Zetaproteobacteria* evolution, ecology and impacts on geochemical cycling. This article was submitted to an online preprint archive (McAllister *et al*. 2018).

## *Zetaproteobacteria*: a novel class of marine Fe(II)-oxidizing bacteria

The discovery of *Zetaproteobacteria* is a story that began decades before the class was proposed. The unusual morphology of biogenic Fe(III) (oxyhydr)oxides have long been used to recognize microbial Fe(II) oxidation in terrestrial environments. The twisted stalks of *Gallionella* and hollow sheaths containing cells of *Leptothrix* were described in terrestrial Fe(II)-rich environments as early as the mid-1800s (Ehrenberg 1838; Kützing 1843). However, Winogradsky (1888) was the first to confirm that *Leptothrix* required Fe(II) for growth, thus linking microbial activity with iron mineral deposition in terrestrial environments (Harder 1919). In the 1980s, similar structures were found in Fe(II)-rich marine environments, including the Red Seamount of the East Pacific Rise, the Explorer Ridge and Loihi Seamount (Fig. S1, Supporting Information) (Alt *et al*. 1987; Alt 1988; Juniper and Fouquet 1988; Karl *et al*. 1988; Karl, Brittain and Tilbrook 1989). This led to the assumption that these structures were made by *Gallionella* and *Leptothrix*, though these organisms were not detected in subsequent studies of marine Fe(II)-oxidizing microbial mats (Fe mats) based on small subunit ribosomal RNA (SSU rRNA, frequently referred to as 16S rRNA) marker gene surveys (e.g. Moyer, Dobbs and Karl 1995; Davis *et al*. 2009; Rassa *et al*. 2009). Instead, Moyer et al. (1995) discovered the first sequence of the novel *Zetaproteobacteria* class, though it was not recognized at the time because there were no isolates or other closely related sequences. The first isolates, *Mariprofundus ferrooxydans* strains PV-1 and JV-1, were obtained from samples collected at Loihi Seamount near Hawaii in 1996–98 (Emerson and Moyer 2002; Emerson *et al*. 2007). Additional surveys from Fe(II)-rich environments provided related 16S rRNA gene sequences (Eder *et al*. 2001; Dhillon *et al*. 2003; Davis *et al*. 2009; Kato *et al*. 2009b), which helped establish the *Zetaproteobacteria* as a monophyletic group within the *Proteobacteria* (Emerson *et al*. 2007). The association of the *Zetaproteobacteria* and Fe(II)-rich marine environments has been strengthened since these initial observations, with continued discovery of *Zetaproteobacteria* within Fe(II)-rich saline environments.

## *Zetaproteobacteria* isolates: model systems for microbial Fe(II) oxidation

The difficulty of culturing FeOB has been one of the main challenges in demonstrating marine microbial Fe(II) oxidation. The first *Zetaproteobacteria* isolates were obtained using liquid and agarose-stabilized gradient tubes and plates designed to provide both Fe(II) and O_2_ in opposing gradients (Emerson and Moyer 2002; Emerson and Floyd 2005). With this setup, Fe(II) is gradually released by dissolution of solid reduced Fe minerals (e.g. Fe(0), FeS, or FeCO_3_) at the bottom of the tube or plate while O_2_ diffuses from the headspace above. These culturing techniques make it difficult to control O_2_ and Fe(II) concentrations. To date, *Zetaproteobacteria* have not been culturable on solid media, so isolation requires serial dilution to extinction, with transfers every ∼2–3 days due to increasing autocatalytic Fe(II) oxidation over time (Lueder *et al*. 2018). In all, these challenges likely account for why so few *Zetaproteobacteria* have been isolated.

Despite these hurdles, *Zetaproteobacteria* representatives from two genera and eight OTUs have been successfully isolated (Table 1). These include seven isolates from microbial mats at Fe(II)-rich hydrothermal vents, and eight from coastal environments. *Mariprofundus ferrooxydans* PV-1 is the type strain of the most frequently isolated genus, and is an obligate neutrophilic autotrophic Fe(II)-oxidizer. All but two other isolates are similarly obligate Fe(II)-oxidizers. These two, *Ghiorsea bivora* TAG-1 and SV-108, are facultative Fe(II)-oxidizers that are also capable of growth by H_2_ oxidation (Mori *et al*. 2017). Except for this instance, isolates vary primarily in their physiological preferences (Table 2), which are related to characteristics of their source environments.

**Table 1.** Isolates of the Zetaproteobacteria and their assigned ZOTUs, with representation in the environment, biomineral and metabolic properties, and references.

Isolate	ZOTU	ZOTU Envir. abund.^a^	Isolation source	Primary biomineral morphology	Fe(II) oxidation	H_2_ oxidation	References
*Mariprofundus ferrooxydans* PV-1	ZOTU11	1.58%	Loihi hydrothermal vents	stalk	X		Emerson and Moyer 2002
*Mariprofundus ferrooxydans* JV-1	ZOTU11		Loihi hydrothermal vents	stalk	X		Emerson and Moyer 2002
*Mariprofundus ferrooxydans* M34	ZOTU11		Loihi hydrothermal vents	stalk	X		McAllister et al. 2011
*Mariprofundus ferrooxydans* SC-2	ZOTU11		Big Fisherman's Cove pyrrhotite colonization	stalk	X		Barco et al. 2017
*Mariprofundus ferrinatatus* CP-8	ZOTU37	0.09%	Chesapeake Bay stratified water column	dreads only	X		Chiu et al. 2017
*Mariprofundus aestuarium* CP-5	ZOTU18	1.49%	Chesapeake Bay stratified water column	dreads only	X		Chiu et al. 2017
*Mariprofundus micogutta* ET2	ZOTU18		Bayonnaise hydrothermal vents	thin filaments	X		Makita et al. 2017
*Mariprofundus* sp. DIS-1	ZOTU18		West Boothbay Harbor mild steel incubation	stalk	X		Mumford et al. 2016
*Mariprofundus* sp. GSB-2	ZOTU23	0.26%	Great Salt Bay salt marsh Fe mat	stalk	X		McBeth et al. 2011
*Mariprofundus* sp. EKF-M39	ZOTU36	0.35%	Loihi hydrothermal vents	stalk	X		Field et al. 2015
*Ghiorsea bivora* TAG-1	ZOTU9	8.42%	MAR hydrothermal vents	none	X	X	Mori et al. 2017
*Ghiorsea bivora* SV-108	ZOTU9		Mariana hydrothermal vents	none	X	X	Mori et al. 2017
Zetaproteobacteria sp. CSS-1	ZOTU14	4.04%	coastal sediment bloodworm microcosm	stalk	X		Beam et al. 2018
Zetaproteobacteria sp. S1OctC	ZOTU3	3.51%	Norsminde Fjord estuary sediments	stalk	X		Laufer et al. 2017
Zetaproteobacteria sp. S2.5	ZOTU3		Kalø Vig beach sediments	stalk	X		Laufer et al. 2017

a Estimates of ZOTU environmental abundance based on 16S rRNA gene surveys (SILVA release 128), including in the estimate counts for instances where a single published sequence represents multiple clones. ZOTU environmental abundance estimates are given once for each ZOTU.

**Table 2.** Growth preferences of Zetaproteobacteria isolates, including optimal growth salinity, temperature, pH and oxygen concentrations.

			Growth salinity (ppt)			Growth temperature (°C)			Growth pH			Growth [O_2_]		
Isolate	ZOTU	Doubling time (h)	Isolation	Range	Opt.	Isolation	Range	Opt.	Isolation	Range	Opt.	Headspace(% O_2_)	Range (µM)	References
*Mariprofundus ferrooxydans* PV-1	ZOTU11	12	35 (ASW)	3.5-35^a^	28-31.5^a^	12-24	10-30	30	6.4-6.5	5.5-7.2	6.2-6.5	1	–	Emerson and Moyer 2002; Emerson et al. 2007
*Mariprofundus ferrooxydans* JV-1	ZOTU11	12	35 (ASW)	–	–	12-24	10-30	30	6.4-6.5	5.5-7.2	6.2-6.5	1	–	Emerson and Moyer 2002; Emerson et al. 2007
*Mariprofundus ferrooxydans* M34	ZOTU11	–	35 (ASW)	–	–	RT	–	–	–	–	–	5-15	–	McAllister et al. 2011
*Mariprofundus ferrooxydans* SC-2	ZOTU11	–	35 (ASW)	–	–	RT	–	–	6.2-6.5	–	–	3.4	–	Barco et al. 2017
*Mariprofundus ferrinatatus* CP-8	ZOTU37	27	18 (SEM)^b^	7-31.5	14-17.5	RT	15-35	25-30	6.2	5.5-8.3	6.9-7.2	1	0.07-1.7	Chiu et al. 2017
*Mariprofundus aestuarium* CP-5	ZOTU18	19.5	18 (SEM)^b^	7-31.5	14-17.5	RT	10-30	20-25	6.2	5.5-8.3	6.9-7.2	1	0.31-2.0	Chiu et al. 2017
*Mariprofundus micogutta* ET2	ZOTU18	24	35 (ASW)	10-40	27.5	25	15-30	25	6.2-6.5	5.8-7.0	6.4	1-3	–	Makita et al. 2017
*Mariprofundus* sp. DIS-1	ZOTU18	–^c^	35 (ASW)	–	–	RT	–	–	6.1-6.4	max 8	–	5-15	max ∼220	Mumford et al. 2016
*Mariprofundus* sp. GSB-2	ZOTU23	13^d^	35 (ASW)	1.75-35	–	RT	–	25	6.1-6.4	max 7.25	–	5-15	–	McBeth et al. 2011
*Mariprofundus* sp. EKF-M39	ZOTU36	–	35 (ASW)	–	–	–	–	–	6.1-6.4	–	–	0^e^	–	Field et al. 2015
*Ghiorsea bivora* TAG-1	ZOTU9	21.8^f^	35 (ASW)	–	–	–	5-30	20	6.5	5.5-7.5	6.5-7.0	0.4-15	–	Mori et al. 2017
*Ghiorsea bivora* SV-108	ZOTU9	20.0^f^	35 (ASW)	–	–	–	5-30	20	6.5	6.0-7.5	6.5-7.0	0.4-15	–	Mori et al. 2017
Zetaproteobacteria sp. CSS-1	ZOTU14	–	35 (ASW)	–	–	RT	–	–	–	–	–	5-10	opt. 60^g^	Beam et al. 2018
Zetaproteobacteria sp. S1OctC	ZOTU3	–	23 (ASW)	6.9-23	–	–	–	–	7.1	–	–	6-10	–	Laufer et al. 2017
Zetaproteobacteria sp. S2.5	ZOTU3	–	23 (ASW)	6.9-23	–	–	–	–	7.1	–	–	6-10	–	Laufer et al. 2017

RT = room temperature; ASW = Artificial Seawater Medium; –  = No data available.

a) Data from Chiu et al. (2017).

b) SEM = Simulated estuary medium (50:50 MWMM:ASW); MWMM = Modified Wolfe's Mineral Medium.

c) Cited as comparable with other FeOB.

d) 13 h doubling time in standard gradient tubes; 7–8 h doubling time on metal coupons.

e) Trace O_2_ was likely introduced into the culture with aerobic vitamin/mineral solutions and mat innoculum.

f) Doubling time on Fe(II) shown; Doubling time on H_2_ was 14.1 h and 16.3 h for TAG-1 and SV-108, respectively.

g) Reported as approximate optimum.

**Figure 1.**
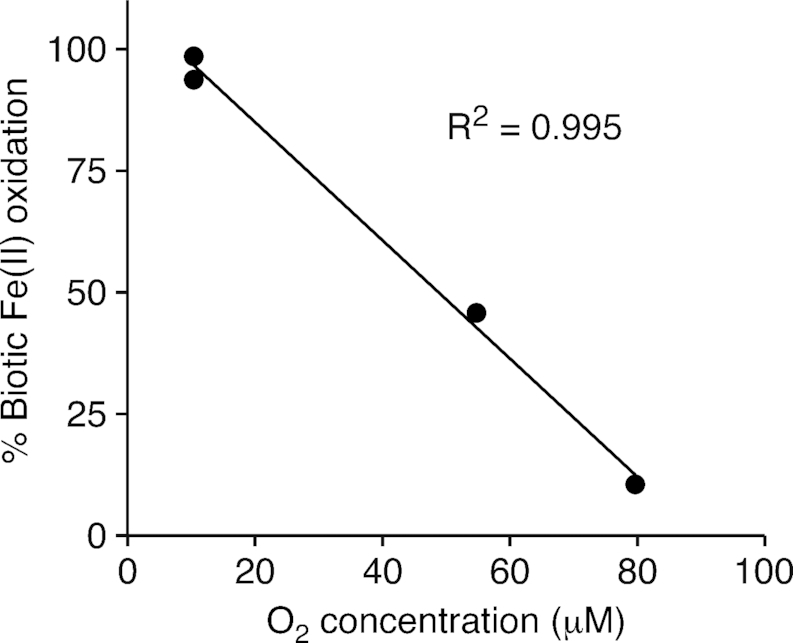
Biotic Fe(II) oxidation rate as a percentage of the total Fe(II) oxidation rate (biotic plus abiotic) at varying oxygen concentrations, using the model *Zetaproteobacteria* isolate, *M. ferrooxydans* PV-1. Range of 6.5 – 6.7 pH for experimental conditions. Further details in *Supplemental Methods*.

*Zetaproteobacteria* isolates are generally microaerophiles originating from oxic-anoxic transition zones, where O_2_ concentrations are low, i.e. micromolar to submicromolar. Abiotic Fe(II) oxidation is slow at these low O_2_ concentrations (Stumm and Lee 1961; Millero, Sotolongo and Izaguirre 1987), which allows the *Zetaproteobacteria* to compete. In terrestrial freshwater circumneutral environments, kinetics experiments near 25°C suggest that biotic Fe(II) oxidation is a significant component of total Fe(II) oxidation below 50 µM and can outcompete abiotic Fe(II) oxidation at 15 µM O_2_ (Druschel *et al*. 2008). However, there are no kinetics data from marine FeOB. To understand the conditions where marine biotic Fe(II) oxidation is competitive, we measured Fe(II) oxidation kinetics using *M. ferrooxydans* PV-1 as a model (see *Supplemental Methods*). With this experiment, we have shown that PV-1 outcompetes abiotic oxidation below 49 µM O_2_, and accounts for up to 99% of the Fe(II) oxidation at 10 µM O_2_ (Fig. 1; Table 3). In cultures of *M. aestuarium* CP-5 and *M. ferrinatatus* CP-8, oxygen concentrations ranged from 0.07–2.0 µM O_2_ within the cell growth band (Chiu *et al*. 2017). This range of O_2_ growth conditions is well below the level at which almost all Fe(II) oxidation was biotic for PV-1, suggesting that many *Zetaproteobacteria* are well adapted to compete and thrive under micromolar and submicromolar O_2_ concentrations. Such low O_2_ concentrations are common within the oxic-anoxic transition zones where the *Zetaproteobacteria* are found (e.g. Chan *et al*. 2016; Field *et al*. 2016).

**Table 3.** Biotic and abiotic Fe(II) oxidation rates of *Mariprofundus ferrooxydans* PV-1 under a range of O_2_ concentrations.

	Fe(II) oxidation rate				
O_2_ conc.	biotic	biotic	abiotic		
µM	µM Fe(II) hr^−1^	µM Fe(II) cell^−1^ hr^−1^	µM Fe(II) hr^−1^	% Biotic Fe(II) oxidation	pH
10.4	21.05	5.06E-04	0.32	98.5	6.63
10.4	24.69	5.53E-04	1.66	93.7	6.70
54.8	33.32	1.00E-03	39.43	45.8	6.67
79.7	5.57	1.25E-04	47.31	10.5	6.50

## Iron biomineral morphologies: form follows function

**Figure 2.**
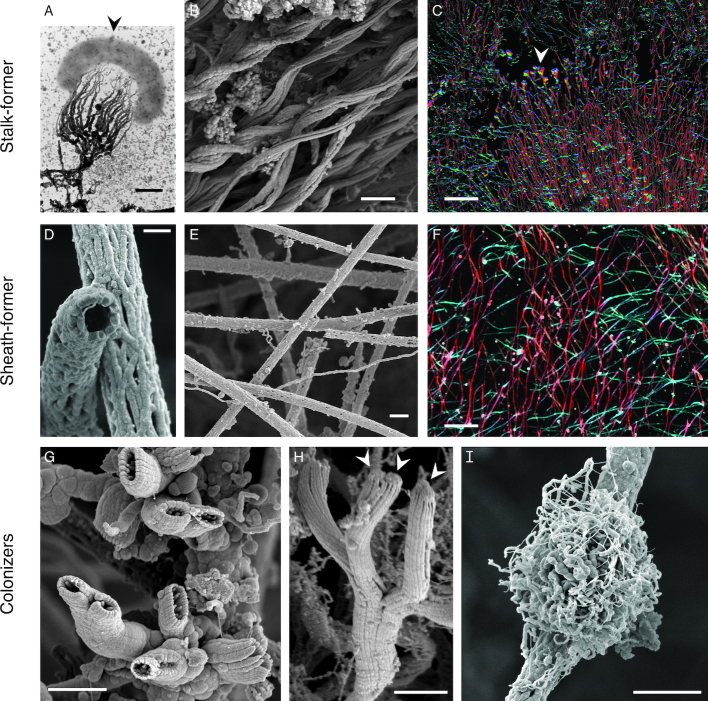
Morphologies of FeOOH biominerals known or suspected to be formed by the Fe(II)-oxidizing *Zetaproteobacteria*. (A–C) Twisted stalks from individual cell to intact Loihi Fe microbial mats. (D–F) Sheaths from individual tubes to intact Loihi mats. (G–I) Fe biominerals attached to stalks and sheaths in Loihi mats. (A) A single *M. ferrooxydans* PV-1 cell (arrow) and its fibrillar stalk. (B) Intact curd-type mats (Fig. 4A) are composed of parallel stalks. (C) Intact mat showing directional stalks that are interrupted (arrow), before biomineral production resumes. See Chan et al. (2016) for details. (D) Hollow sheaths formed by the *Zetaproteobacteria*. (E) Intact veil-type mats (Fig. 4B) are composed of sheaths. (F) Zoomed out intact mat with multiple sets of sheaths oriented in different directions. (C,F) Color corresponds with filament direction/orientation. (G–H) Short, Y-shaped tubular biominerals formed by *Zetaproteobacteria*. (H) Arrows show attached cells. (I) *Siderocapsa*-like nest-type biominerals; it is not known if they are formed by the *Zetaproteobacteria*. Scale bars: 0.5 µm (A,D), 2 µm (B,E,G–I), 100 µm (C,F). Images reproduced with permission: (A) from Chan *et al*. (2011), (F, H) from Chan *et al*. (2016). (B–I) from the samples described in Chan *et al*. (2016). (D, I) imaged on JEOL-7200 field emission SEM.

*M. ferrooxydans* PV-1 has been a model system for biomineralization by an obligate Fe(II)-oxidizer. PV-1 cells form a twisted stalk (Fig. 2A–C), so similar to the one formed by the terrestrial Fe(II)-oxidizer *Gallionella ferruginea* that it could be mistaken for a *Gallionella* stalk (Emerson and Moyer 2002). The stalk consists of individual filaments made of nanoparticulate Fe(III) oxyhydroxides and acidic polysaccharides, controlling Fe mineral growth near the cell surface (Chan *et al*. 2011). Stalk growth was measured to be 2.2 µm length h^−1^, or nearly 5x the width of a PV-1 cell per hour (Chan *et al*. 2011). The combination of this directed mineralization and a near-neutral cell surface charge explains how the cell remains remarkably free of encrustation (Saini and Chan 2012). These encrustation avoidance mechanisms are important for any Fe(II)-oxidizing microbe to avoid cell death by Fe mineral growth inside and outside the cell.

**Figure 3.**
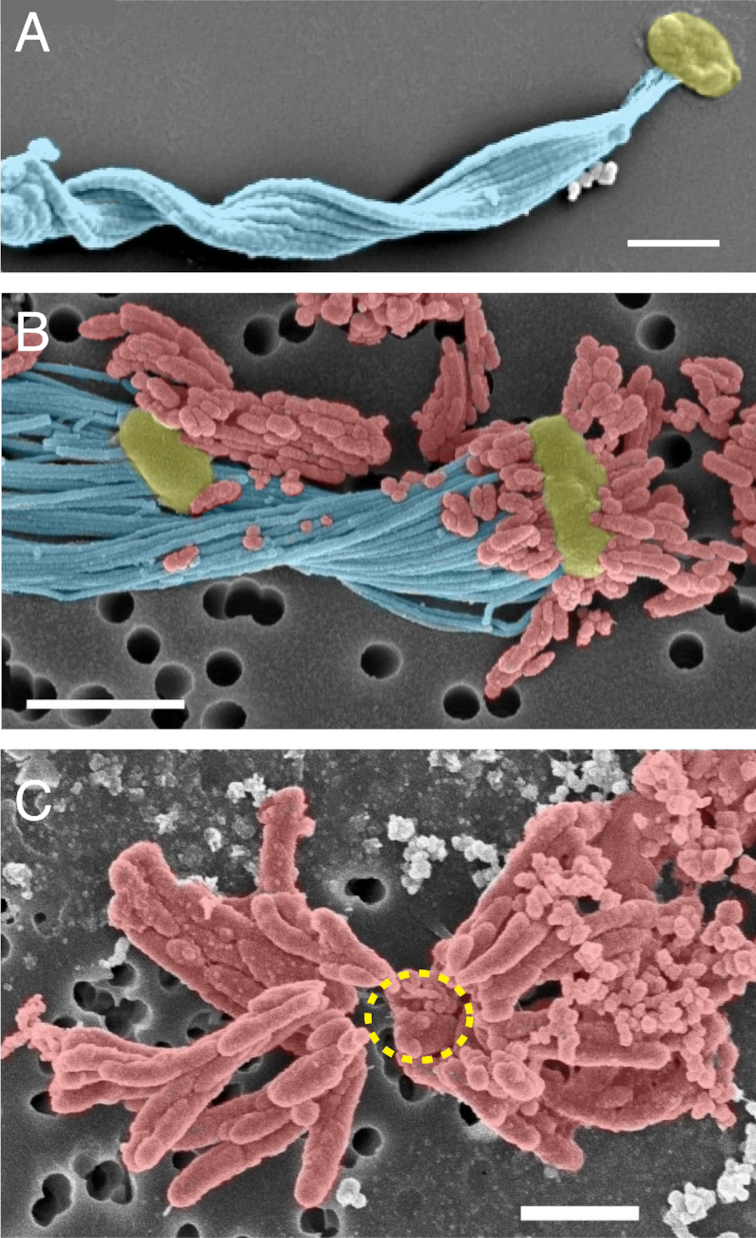
Colorized SEM images of stalk and dread biominerals of selected marine and freshwater FeOB. (A) Twisted stalk (blue) formed by a *M. ferrooxydans* PV-1 cell (yellow). Most *Zetaproteobacteria* isolates form stalks. (C) In contrast, *M. aestuarium* CP-5 and *M. ferrinatatus* CP-8 produce only short dreads (red), which are easily shed from the cell (inferred cell position indicated by dashed yellow line). (B) Stalk (blue) and dreads (red) of the freshwater Betaproteobacteria FeOB *Ferriphaselus* sp. R-1 resembled the structures formed by marine FeOB. Scale bars = 1 µm. Images reproduced with permission: (A) from Chan *et al*. (2016), (B) from Kato *et al*. (2015b), (C) from Chiu *et al*. (2017).

While most *Zetaproteobacteria* isolates form a stalk, some make other biomineral morphologies (Table 1; Fig. 3). *Mariprofundus ferrinatatus* CP-8 and *M. aestuarium* CP-5 form shorter filaments that resemble the dreadlock hairstyle (Fig. 3C; Chiu *et al*. 2017). Dreads were originally observed in terrestrial FeOB *Gallionellaceae Ferriphaselus spp*., which makes both stalk and dreads (Fig. 3B; Kato *et al*. 2015b). In both *Ferriphaselus* and the CP strains, the dreads are shed from cells. This suggests that dreads and similar structures are used specifically to avoid encrustation, whereas the stalk has other functions. PV-1 cells use the stalk as a holdfast to anchor the cell to surfaces (Krepski *et al*. 2013). As the cell oxidizes Fe(II) and produces new stalk, the cell moves forward, leaving stalk behind. Since the stalk is rigid and anchored, this is a means of motility. Experiments in controlled Fe(II) and O_2_ gradients showed that PV-1 cells use their stalks to position themselves at an optimum position within that gradient, often forming filaments oriented toward higher O_2_ (Krepski *et al*. 2013).

**Figure 4.**
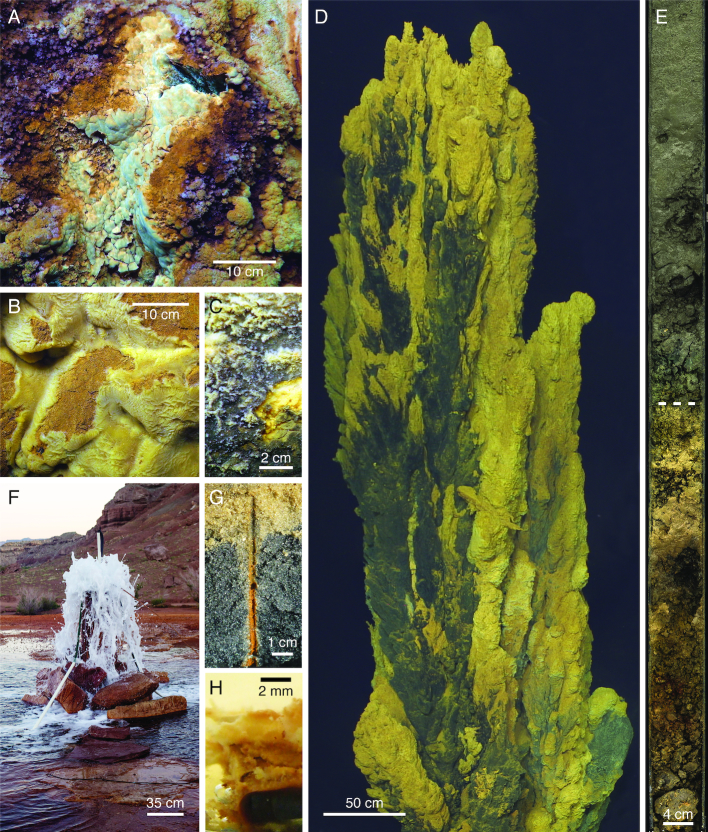
Photographs of *Zetaproteobacteria* habitats. (A–D) Marine hydrothermal vent mats, where *Zetaproteobacteria* have been found in highest abundance. (A) Curd-type and (B) veil-type Fe mats, from Loihi Seamount. (C) Mn-crusted Fe mat from the Ula Nui site, Loihi. Fe mat visible under broken surface (bottom right). (D) Fe mats on the Golden Horn Chimney, at the Urashima vent site, Mariana Trough. (E) Transition from reduced to Fe(III) (oxyhydr)oxide-stained marine sediments (dashed line) in 26 m below seafloor core from the hydrothermal circulation cell of Iheya North vent field, Okinawa Trough. See Takai *et al*. (2012) for details. (F) Terrestrial saline CO_2_-rich spring at Crystal Geyser, UT, USA. (G) Fe(III) (oxyhydr)oxide-coated worm burrows from the beach at Cape Shores, DE, USA. (H) Mild steel corrosion biofilm formed by isolate *M*. sp. GSB-2. Original photography reproduced with permission: (H) by Joyce M. McBeth, (F) by Chris T. Brown.

In the environment, such oriented filaments are common. At Loihi Seamount, curd-type mats (cohesive Fe mats with a bumpy surface reminiscent of cheese curds) often form directly above a vent orifice (Fig. 4A) (Chan *et al*. 2016). Micrographs of intact curd mats showed centimeters-long, highly directional twisted stalks forming the mat architecture (Fig. 2A-C). These stalks record the synchronous movements of a community of cells all growing and twisting in the same direction, as well as shifts in directionality in response to changes in the environment (Chan *et al*. 2016). The mechanism by which these cells actively control their directionality through stalk production is currently unknown.

Beyond stalks, Loihi Seamount also hosts sheath-rich veil-type mats, which form millimeters-thick Fe mat draped over rock or older Fe mat in diffuse venting environments (Fig. 4B). These mats are created by organisms that form hollow Fe(III) (oxyhydr)oxide sheaths (Fig. 2D–F), similar to those produced by the terrestrial *Betaproteobacteria Leptothrix*. In the marine environment, however, these sheaths are formed by *Zetaproteobacteria* (Fleming *et al*. 2013), informally called zetathrix. From studies based on the terrestrial *Leptothrix*, sheaths function similarly to stalks, with tens of cells producing a single sheath and leaving it behind as the cells move forward (Chan *et al*. 2016). In Loihi Seamount intact veil mats, sheaths also leave a record of highly directional growth, despite oxygen profiles of these mats showing a shallow O_2_ gradient with O_2_ present throughout the mat (Chan *et al*. 2016). In both curd- and veil-type mats, *Zetaproteobacteria* work together to form a highly porous and fluffy mat almost completely composed of biomineral filaments formed by cells, making these Fe mats different from other commonly studied mats or biofilms, which feature cells embedded in an exopolysaccharide matrix. The biomineral filaments forming the structure of the mat also frequently have Fe biominerals attached to them, suggesting FeOB also colonize the mat interior (Fig. S1, Supporting Information). Short branching hollow tubes are formed by the *Zetaproteobacteria* (Fig. 2G,H) (Emerson *et al*. 2017) informally called “y-guys.” However, the organisms forming other Fe biominerals have yet to be identified, including nest-like structures reminiscent of freshwater *Siderocapsa*-like organisms (Fig. 2I) (Emerson, Fleming and McBeth 2010). The range of biomineral morphologies is related to differing biomineral functions, which likely correspond to different geochemical/physical niches within the Fe mat habitat.

## Habitats of the *Zetaproteobacteria*

The Zetaproteobacteria are found in a variety of Fe(II)-rich habitats globally. The detection or observation of Zetaproteobacteria in these habitats is based almost exclusively on the distribution of the 16S rRNA gene. This gene is by far the most frequently used in microbial surveys, making it the best means of comparing Zetaproteobacteria ecology across studies. Isolation, direct observation using fluorescent probes, and metagenomic reconstruction have also been used to identify the Zetaproteobacteria, though in only a few instances, as noted.

### Loihi Seamount hydrothermal vents: a *Zetaproteobacteria* observatory

Most of what we know about *Zetaproteobacteria* is based on work at the Loihi Seamount, a Hawaiian submarine volcano, from long-term studies including the Iron Microbial Observatory (FeMO). Loihi Seamount is an ideal habitat for *Zetaproteobacteria*, with hydrothermal fluids rich in CO_2_ (up to 303 mmol/kg) and Fe(II) (up to 934 µM), and low in sulfide (<50 µM in vent fluids; undetectable in Fe mats) (Karl *et al*. 1988; Sedwick, McMurtry and Macdougall 1992; Glazer and Rouxel 2009). Background seawater oxygen concentrations are ∼50 µM at the summit of Loihi Seamount, due to its location within the oxygen minimum zone (Glazer and Rouxel 2009). At the base of Loihi Seamount, the Ula Nui site has higher ambient O_2_ concentrations (145 µM), but lower venting temperatures (1.7°C average compared to ∼42°C average at the summit) (Edwards *et al*. 2011). Low temperatures and low ambient O_2_ concentrations favor biotic Fe(II) oxidation by reducing the abiotic rate at and below the mat surface (Millero, Sotolongo and Izaguirre 1987; Emerson *et al*. 2015). Thus, the conditions at Loihi Seamount have favored the growth of Fe microbial mats ranging from centimeters to meters thick and up to 15 km^2^ (Fig. 4A–C) (Edwards *et al*. 2011; Chan *et al*. 2016). The extensive Fe mats at Loihi Seamount may reflect years- to decades-long stable Fe mat production by the *Zetaproteobacteria*, based on productivity estimates (Chan *et al*. 2016; Emerson *et al*. 2017).

Loihi Seamount studies have provided the cornerstones of *Zetaproteobacteria* ecology. Since the discovery of *Zetaproteobacteria* in the 1990s (Moyer, Dobbs and Karl 1995; Emerson and Moyer 2002), five research expeditions from 2006–2013 have focused on *Zetaproteobacteria* succession, niche and species diversity, and genetic potential. Colonization experiments over 4–10 days showed that *Zetaproteobacteria* prefer low- to mid-temperature (from 22–60°C, average 40°C) Loihi hydrothermal vents (Rassa *et al*. 2009). This preference was reflected in longer term observations following the 1996 Loihi eruption, which showed *Zetaproteobacteria* increasing in abundance as high temperature vents cooled to pre-eruption temperatures and transitioned from sulfide-rich to Fe(II)-rich fluids (Davis *et al*. 2005; Moyer *et al*. 2007; Glazer and Rouxel 2009; Emerson and Moyer 2010). The bulk of the omic information on *Zetaproteobacteria* originates from Loihi Seamount, with the first isolate genome, single cell genomes, metagenome and proteome all from Loihi sources (Singer *et al*. 2011, 2013; Barco *et al*. 2015; Field *et al*. 2015).

### Other hydrothermally influenced habitats

Beyond Loihi, *Zetaproteobacteria* are hosted by many other hydrothermal systems. Extensive Fe mats form around vents at seamounts and island arc systems (Fig. 4D) (Kato *et al*. 2009a; Emerson and Moyer 2010; Makita *et al*. 2016; Bortoluzzi *et al*. 2017; Hager *et al*. 2017). However, Fe mats have also been found at spreading ridge systems, within diffuse flow at the periphery of high-temperature chimneys and vents (Dekov *et al*. 2010; Breier *et al*. 2012; Scott *et al*. 2015; Vander Roost, Thorseth and Dahle 2017). Most hydrothermal Fe mats consist of biomineral morphologies similar to those at the Loihi Seamount (twisted stalks, sheaths, y-guys, etc.) (Breier *et al*. 2012; Scott *et al*. 2015). However, mat textures and lithification can vary as a function of geochemistry (e.g. Mn, Si concentration) and rates of hydrothermal discharge (Li *et al*. 2012; Johannessen *et al*. 2017).

*Zetaproteobacteria* are also found in the marine subsurface. There, oxygenated seawater can mix with anoxic Fe(II)-rich fluids, providing a favorable environment for Fe(II) oxidation. *Zetaproteobacteria* have been observed by both 16S rRNA gene surveys and metagenomic reconstruction up to 332 meters below the sea floor, within both hydrothermal recharge and cold oxic circulation cells (Fig. 4E) (Yanagawa *et al*. 2013; Meyer *et al*. 2016; Tully *et al*. 2018). In many near surface sediments, shallow mixing introduces O_2_ into an Fe(II)-rich environment, leading to abundant *Zetaproteobacteria* populations (Davis *et al*. 2009; Kato *et al*. 2009b; Handley *et al*. 2010; Gonnella *et al*. 2016). As hydrothermal systems age and cool, basalts and the minerals within inactive sulfide mounds can also serve as Fe(II) sources for *Zetaproteobacteria* (Sylvan, Toner and Edwards 2012; Kato *et al*. 2015a; Henri *et al*. 2016; Barco *et al*. 2017). These studies show that the *Zetaproteobacteria* are abundant members of shallow and deep marine subsurface environments, one of the largest underexplored habitats in the oceans.

### Coastal and terrestrial habitats

*Zetaproteobacteria* have only recently been discovered in coastal and terrestrial environments. Colonization experiments showed that *Zetaproteobacteria* biofilms grow on Fe(II) released from mild and carbon steel that is commonly used in ships and docks, suggesting that these FeOB contribute to corrosion (Fig. 4H) (Dang *et al*. 2011; McBeth *et al*. 2011; Lee *et al*. 2013; McBeth and Emerson 2016; Mumford, Adaktylou and Emerson 2016). [For a review on the role of FeOB in biocorrosion, see Emerson 2019.] Fe(II) can also come from natural sources in coastal environments, originating from mineral weathering and Fe(III) reduction and transported in anoxic groundwater. Fe redox cycling at the oxic-anoxic transition zone of stratified estuaries can support the growth of *Zetaproteobacteria*, as evidenced by the isolation of *M. ferrinatatus* CP-8 and *M. aestuarium* CP-5 (Field *et al*. 2016; Chiu *et al*. 2017). In near shore sediments, Fe(II)-rich groundwater can support microbial communities with *Zetaproteobacteria* at the sediment surface (Rubin-Blum *et al*. 2014; Laufer *et al*. 2016; Hassenrück *et al*. 2016; Otte *et al*. 2018). Also in these sediments, bioturbation from plant roots and animal burrows provides conduits of O_2_ to this Fe(II)-rich groundwater. Biotic and abiotic Fe(II) oxidation in these environments leads to the formation of Fe(III) (oxyhydr)oxides, which coat sands, salt grass and mangrove roots and burrows (Fig. 4G) (Taketani *et al*. 2010; McBeth *et al*. 2011; McAllister *et al*. 2015; Beam *et al*. 2018). Beam et al. (2018) found the abundance of *Zetaproteobacteria* within Fe(III) oxide-coated worm burrows to be an order of magnitude higher than surrounding bulk sediment, suggesting that *Zetaproteobacteria* growth and biotic Fe(II) oxidation can be favored in these bioturbated sediments. The Fe(III) (oxyhydr)oxides produced in these environments can sequester toxins that adsorb to the mineral surface (Charette, Sholkovitz and Hansel 2005). Thus, FeOB activity could affect coastal water quality.

*Zetaproteobacteria* have generally been considered marine FeOB, detected at salinities up to 112 ppt in hypersaline brines (Eder *et al*. 2001; Guan *et al*. 2015). Their occurrence in coastal environments provides the opportunity to delineate their minimum salinity requirements. McBeth et al. (2013) surveyed Fe mats along the tidal Sheepscot River, Maine, as it entered the estuary, finding that *Zetaproteobacteria* only appeared in environments with 5 ppt salinity or higher. This explains why *Zetaproteobacteria* are not commonly found nor expected to be found in most terrestrial environments.

Thus, it was interesting and novel to find abundant populations of *Zetaproteobacteria* in CO_2_-rich terrestrial springs. Surveys of the 16S rRNA genes from carbonic springs at Tierra Amarilla Spring, New Mexico (∼9 ppt salinity) revealed a microbial population up to one third *Zetaproteobacteria* (Colman *et al*. 2014). Similarly, 16S rRNA gene and metagenomic work at the CO_2_-rich Crystal Geyser, Utah, (∼11-14 ppt salinity; Fig. 4F) found the *Zetaproteobacteria* to be both abundant and consistently present over a year of observation (Emerson *et al*. 2016; Probst *et al*. 2017, 2018). These springs represent the first habitat with abundant populations of both *Zetaproteobacteria* and *Betaproteobacteria* FeOB (*Gallionellaceae*), whose abundance is likely driven by cycles of freshwater and saline subsurface groundwater mixing (Probst *et al*. 2018). The work at Crystal Geyser has produced full-length 16S rRNA gene sequences and the only terrestrial *Zetaproteobacteria* genomes (Emerson *et al*. 2016; Probst *et al*. 2017, 2018). Our analysis of 16S rRNA gene phylogenetic placement and genomic clustering (by average nucleotide identity) suggests that *Zetaproteobacteria* populations in terrestrial subsurface environments are primarily novel and deeply branching *Zetaproteobacteria* (see further discussion of habitat selection and niche below).

### Common habitat characteristics

In all, *Zetaproteobacteria* have been found in habitats sharing the following characteristics: 1) brackish to hypersaline water, 2) a supply of Fe(II) and 3) predominantly micro-oxic conditions. These conditions are widespread and found in diverse habitats, likely supplying multiple niches for the diversification and evolution of the *Zetaproteobacteria*.

## *Zetaproteobacteria* diversity

**Figure 5.**
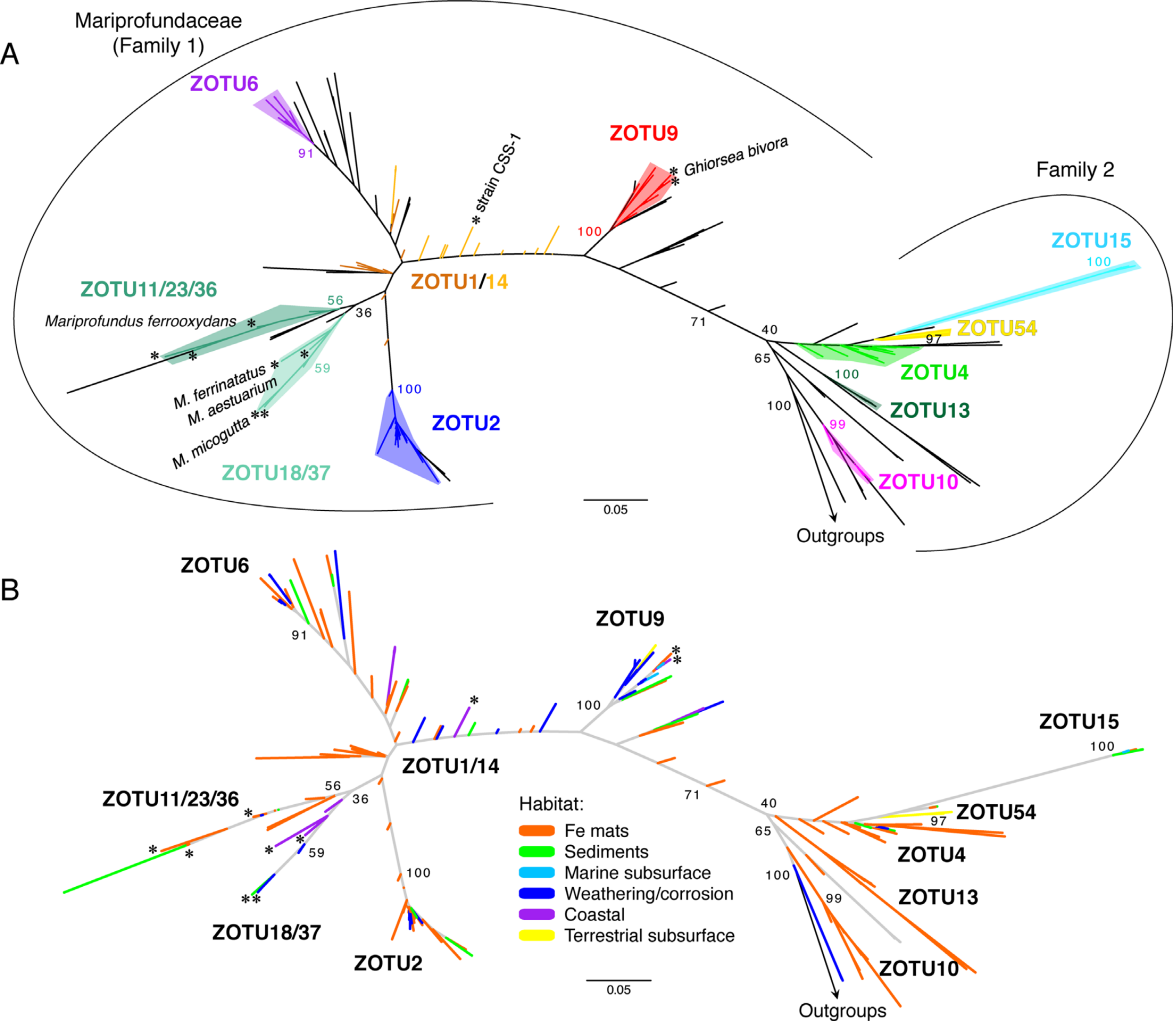
Maximum likelihood phylogenetic tree showing *Zetaproteobacteria* 16S rRNA gene diversity, (A) colored by ZOTU and (B) colored by habitat type where the sequences were sampled. A total of 59 ZOTUs have been classified, though only the most frequently sampled are shown in the figure above. ZOTUs 1 and 14 are poorly resolved phylogenetically by the 16S rRNA gene. Published isolates of the *Zetaproteobacteria* are starred and labeled. Phylogenetic trees were colored automatically using Iroki (Moore *et al*. 2018). A rectangular version of this tree is represented in Fig. S2 (Supporting Information), which also includes the habitat information for each sequence.

**Figure 6.**
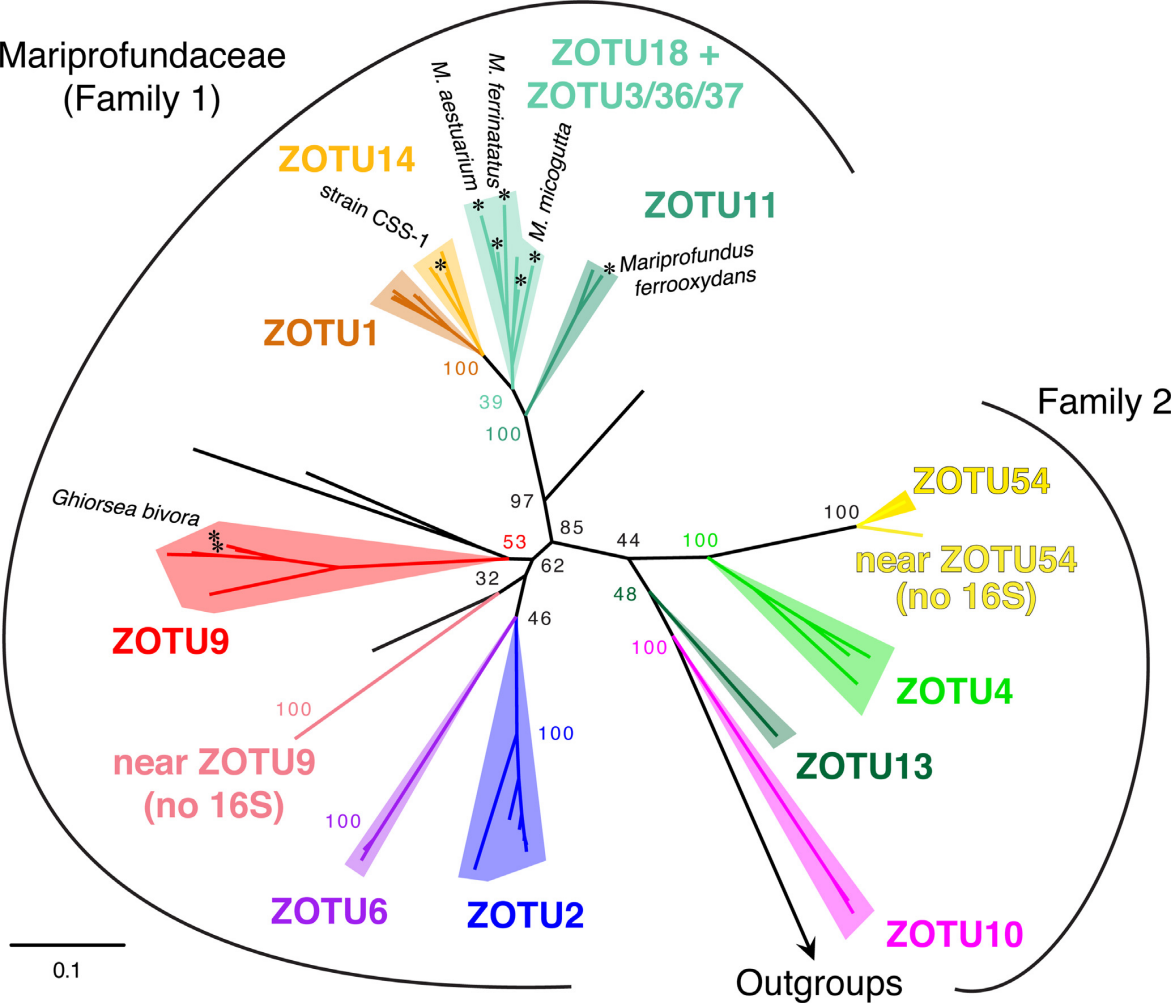
Maximum likelihood phylogenetic tree showing a more robust phylogenetic placement of ZOTUs based on the concatenated alignments of 12 ribosomal proteins. Data from isolate, SAG and MAG genomes (see *Supplemental Methods*). In this tree, ZOTUs 1 and 14 are monophyletic entities. Taxa near ZOTU9 and near ZOTU54 are only represented by genomes; these clades have only been found in the terrestrial CO_2_-rich spring waters of Crystal Geyser, UT.

*Zetaproteobacteria* diversity has been defined using 16S rRNA gene *Zetaproteobacteria* operational taxonomic units (ZOTU; 97% similarity), based on sequences from isolates and environmental samples (Table 1; Dataset S1, Supporting Information). Since their initial description, the *Zetaproteobacteria* class has remained a robust taxonomic group within the *Proteobacteria* (Hug *et al*. 2016; Parks *et al*. 2018). A systematic analysis of 227 *Zetaproteobacteria* full-length 16S rRNA gene sequences yielded 59 ZOTUs (McAllister, Moore and Chan 2018), an increase from 28 ZOTUs in 2011 (McAllister *et al*. 2011). The majority of these ZOTUs are contained within two families of the *Zetaproteobacteria*, based on sequence similarity (Fig. 5A, Fig. 6). Fig. 5 shows key ZOTUs, which are frequently sampled and abundant in the environment and are primarily distinct monophyletic taxonomic groups by 16S rRNA gene (see detail in Fig. S2, Supporting Information). These 15 ZOTUs represent 83% of sequences found in the environment. ZOTUs 1 and 14 are the one exception to monophyly by the 16S rRNA gene, yet do form distinct monophyletic groups in a concatenated tree of 12 ribosomal proteins (Fig. 6; see *Supplemental Methods*). ZOTUs that include isolates represent only 20% of environmental sequences (Table 1), showing that the *Zetaproteobacteria* are largely uncultivated.

In order to compare *Zetaproteobacteria* diversity across studies, we used ZetaHunter, a classification pipeline designed to rapidly and reproducibly classify ZOTUs (McAllister, Moore and Chan 2018). We classified publicly available *Zetaproteobacteria* full- and partial-length 16S rRNA gene sequences from SILVA (Glöckner *et al*. 2017) and Integrated Microbial Genomes (Chen *et al*. 2017), and included data from NCBI SRA (Leinonen, Sugawara and Shumway 2011) as organized by the Integrated Microbial Next Generation Sequencing platform (Lagkouvardos *et al*. 2016) (total of 1.2 million sequences from 93 studies; summary of samples in Dataset S1; see *Supplemental Methods*). This work provided the basis for the habitat and diversity analysis below, while also allowing us to correct previous ZOTU assignments (Table S1, Supporting Information).

### Connecting *Zetaproteobacteria* diversity, habitat and niche

*Zetaproteobacteria* diversity is likely primarily driven by the variety of niches they inhabit. A niche is the set of conditions favorable for growth, which are further influenced, or partitioned, by inter- and intra-species population dynamics in the environment (Holt 2009). A challenge in microbial ecology is to tease apart the niche of an organism through sampling at the appropriate spatial and temporal resolution. For most *Zetaproteobacteria* environments, we lack the highly resolved chemical and spatial information to describe niches. However, we can look for patterns of associations between different *Zetaproteobacteria* and their habitats to understand where and the extent to which *Zetaproteobacteria* niches may overlap.

Each habitat displayed distinct and abundant ZOTUs, indicating that habitats can host a specific set of niches that support these ZOTUs (Table 4; Dataset S1, Supporting Information). In particular, dominant ZOTUs differ between habitat types, suggesting that each habitat has a set of dominant niches that favor the growth of those particular ZOTUs. ZOTU54 is a striking example of a dominant ZOTU clearly successful within the terrestrial subsurface fluid environment. ZOTU54 is a deep-branching ZOTU that is primarily limited to this environment (Colman *et al*. 2014; Emerson *et al*. 2016; Probst *et al*. 2017, 2018). The distribution of ZOTU54 suggests that it is endemic or adapted to thrive within terrestrial carbonic Fe(II)-rich springs. However, while it is frequently found at high abundance in terrestrial springs, ZOTU54 is also found in other habitats at very low abundance, including hydrothermal Fe mats (Dataset S1, Supporting Information). In fact, many ZOTUs span habitats (Fig. 5B; Dataset S1, Supporting Information), suggesting that similar niches supporting these ZOTUs can exist in multiple habitats.

**Table 4.**
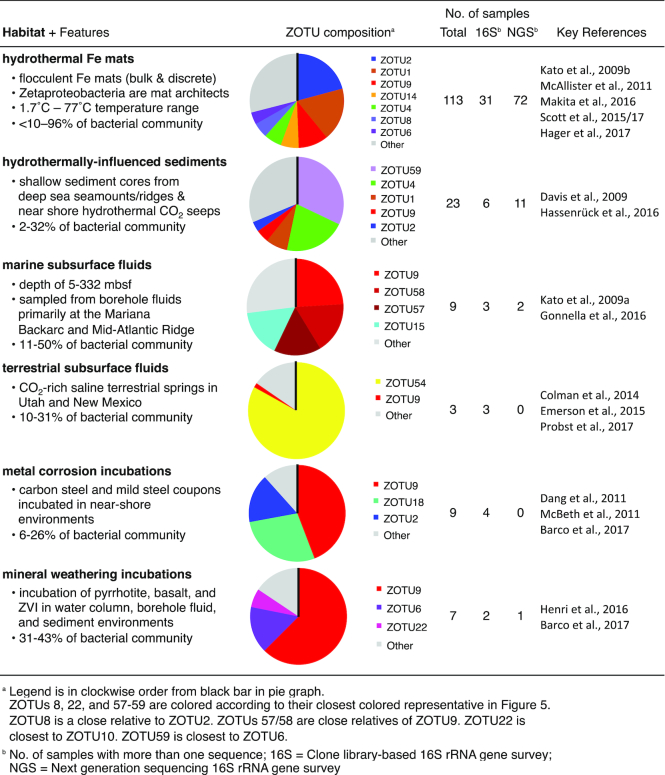
Summary of the habitats where the Zetaproteobacteria are found in high abundance using data from Dataset S1 (Supporting Information).

**Figure 7.**
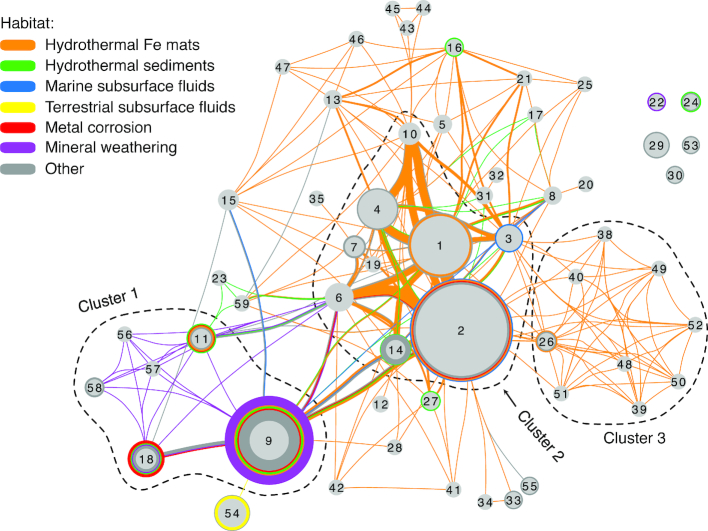
*Zetaproteobacteria* OTU network showing the association of known ZOTUs within the specified habitats. Lines connect ZOTUs that are found in the same sample, with thickness representing the frequency of that association in multiple samples. Colored circles surrounding ZOTU nodes show samples where only a single ZOTU was found. ZOTU nodes are sized according to their environmental abundance. Placement of ZOTUs in the network was determined automatically, based on the frequency of co-occurrence (Cytoscape's edge-weighted, spring embedded layout). Dotted lines denote ZOTU clusters common to the following habitats: Cluster 1, metal corrosion and mineral weathering; Cluster 2, ubiquitous Fe mats; Cluster 3, Mariana Trough Fe mats. Isolate sequences are not shown. Dataset based on SILVA release 128.

Next, we looked for patterns in ZOTU associations with each other, mapping connections in a ZOTU network (Fig. 7). This network shows which ZOTUs are found in isolation and which co-occur, with connections drawn between ZOTUs if they are found in the same sample. Multiple observations of the same connection result in a thicker line, showing the strength of those connections. Further, the network layout is based on the frequency of co-occurrence, so when two ZOTUs commonly co-occur, they are closer together. Most ZOTUs co-occur with others (Fig. 7), and these connections are not random. Some ZOTUs co-occur more frequently, forming clusters of interconnected ZOTU nodes (Clusters 1–3, Fig. 7). The most abundantly sampled ZOTUs form a central cluster (Cluster 2), sharing a common set of niches most frequently sampled in the hydrothermal Fe mat environment. Cluster 3 centers around ZOTUs found together in hydrothermal Fe mat samples from the Mariana Arc, but which are not common in other environments. Cluster 1 is dominated by ZOTUs from samples associated with metal corrosion and mineral weathering, which suggests that these habitats host niches distinct from those in hydrothermal Fe mat habitats. Overall, these clusters highlight ZOTUs with niches that frequently overlap, suggesting those niches, and thus the growth requirements of these ZOTUs, are compatible.

A combination of ZOTU environmental distribution, habitat characteristics and isolate physiology can help us better understand a particular ZOTU's niche. Here, we use this approach to describe ZOTU9, as an example. This ZOTU is a key player in marine subsurface fluids, metal corrosion and mineral weathering habitats (see Cluster 1, Fig. 7; Table 4). In mineral weathering habitats, ZOTU9 is frequently the only ZOTU (visualized as a colored circle around the ZOTU node; Fig. 7). For example, ZOTU9 was the only *Zetaproteobacteria* detected within a basaltic glass weathering enrichment, making up 39% of the bacterial community by 16S rRNA gene sequencing estimates (Henri *et al*. 2016). These habitat associations suggest that growth of ZOTU9 is favored when metal corrosion or mineral weathering is a source for Fe(II). This association likely relates to these sources producing H_2_ as well as Fe(II). Metals composed of zero valent Fe produce H_2_ as a byproduct of anaerobic corrosion (Matheson and Tratnyek 1994). Fe minerals can be a source of H_2_ because they catalyze hydrolysis and induce radiolysis of water (Bach and Edwards 2003; Dzaugis *et al*. 2016). The production of H_2_ is known to benefit some members of ZOTU9, including the Fe(II)- and H_2_-oxidizing isolates *Ghiorsea bivora* TAG-1 and SV-108 (Mori *et al*. 2017). The genetic machinery required for H_2_ oxidation has also been found in two ZOTU9 single amplified genomes (Field *et al*. 2015; Scott *et al*. 2015), which suggests that H_2_ oxidation may be a feature of other members of ZOTU9, though not necessarily all (since ZOTUs are based on 97% 16S rRNA gene similarity). From these observations, we conclude that both Fe(II) and H_2_ may play a central role in the niche of ZOTU9 and the habitats where it can be found. By combining isolate physiology with habitat distribution patterns, we can identify key features of a ZOTU's niche.

### Spatial and taxonomic resolution in *Zetaproteobacteria* ecology

Hydrothermal Fe mats have opposing gradients of Fe(II) and O_2_ and a complex internal structure (e.g. Fig. S1, Supporting Information) (Glazer and Rouxel 2009; Chan *et al*. 2016). This heterogeneity leads to multiple niches at small spatial scales, suggesting that high-resolution sampling could help us better understand ZOTU niches in this habitat. Initial bulk techniques for sampling collected liters of mat material, and a single sample could contain all major ZOTUs (McAllister *et al*. 2011). Therefore, new collection devices were engineered to sample small volumes (50–75 mL) at centimeter spatial resolution (Breier *et al*. 2012). From these discretely sampled Fe mats, we increased the resolution of our ZOTU network (Fig. S3, Supporting Information). Of the 29 ZOTUs found within Fe mat habitats, 17 showed a preference for a specific Fe mat type. However, the high co-occurrence of abundant ZOTUs within a single sample remained, even when considering more highly resolved sampling (Fig. S3, Supporting Information). This result suggests that these ZOTUs share compatible niches at the centimeter scale in Fe mat habitats.

Taxonomic resolution can also affect our understanding of *Zetaproteobacteria* ecology through the lumping or splitting of ecologically-distinct groups. The ZOTU classification may in certain cases be too coarse, representing multiple related populations that have different niches. For example, Scott et al. (2017) found multiple oligotypes (ecological units defined by informative sequence variability) within each ZOTU. While multiple oligotypes do not necessarily suggest each has a distinct niche, for ZOTU6, only one oligotype differed in abundance over a transect approaching the hydrothermal vent orifice. This abundance change suggested that a subpopulation of ZOTU6 prefers higher flow conditions, warmer temperatures and/or the differing geochemistry found near the vent (Scott, Glazer and Emerson 2017). Results like this warrant a more resolved *Zetaproteobacteria* taxonomy, which could be aided by whole genome comparisons.

## Using genomics to understand metabolic potential and niche

Here, we use Zetaproteobacteria genomes to understand metabolic potential and niche, though interpretations are subject to genome completeness and representation of Zetaproteobacteria diversity. Almost all Zetaproteobacteria isolates have high-quality genomes, greater than 99% complete. However, these isolates represent a small portion of Zetaproteobacteria diversity, requiring genomes from single cells and metagenomes (SAGs and MAGs) to better understand their overall metabolic potential. In most cases, these genomes are much less complete, ranging from <10% to 83% completeness (average 46%) for the SAGs (Field et al. 2015) and from <10% to 100% completeness (average 75%) for the MAGs (Fullerton et al. 2017; Probst *et al*. 2017). Thus, gene presence is more informative than absence in the SAGs and MAGs.

### Carbon fixation

All *Zetaproteobacteria* isolates are obligate autotrophs, using the Calvin-Benson-Bassham (CBB) cycle to fix carbon. Similarly, all ZOTUs sampled to date have the ribulose-1,5-bisphosphate carboxylase oxygenase gene (RuBisCO; key enzyme in the CBB cycle), suggesting carbon fixation by this pathway is a shared capability across the class. The isolates of *Mariprofundus ferrooxydans*, strains PV-1, JV-1 and M34, all encode the genes for both Form I (O_2_-insensitive) and Form II (O_2_-sensitive) RuBisCO (Singer *et al*. 2011; Fullerton, Hager and Moyer 2015). Similarly, both forms are encoded by *Mariprofundus*sp. DIS-1, which was specifically isolated to be more aerotolerant (Mumford, Adaktylou and Emerson 2016). However, all other isolates and most *Zetaproteobacteria* SAG and MAG genomes only encode Form II RuBisCO (Field *et al*. 2015; Fullerton *et al*. 2017; Probst *et al*. 2017), suggesting most *Zetaproteobacteria* are specifically adapted to lower O_2_ concentrations.

### Energy metabolism: are all *Zetaproteobacteria* Fe(II)-oxidizers?

The *Zetaproteobacteria* are often associated with high Fe(II) environments, and all isolates of the *Zetaproteobacteria* are capable of Fe(II) oxidation. These observations have led to the current assumption that all *Zetaproteobacteria* are capable of Fe(II) oxidation. To test this assumption, we first have to understand the mechanism of Fe(II) oxidation in the marine environment.

Initial genome analysis of PV-1 led to the proposal of the alternative complex III (ACIII) as part of an iron oxidase complex (Singer *et al*. 2011). Follow-up studies later changed this model, suggesting ACIII was involved in reverse electron transport (Singer *et al*. 2013; Barco *et al*. 2015; Kato *et al*. 2015b). However, Field et al. (2015) and Chiu et al. (2017) isolated *Zetaproteobacteria* isolates that lacked ACIII but were still capable of Fe(II) oxidation. Furthermore, only 2 of 23 *Zetaproteobacteria* SAGs have the ACIII gene, and these 23 SAGs represent the majority of *Zetaproteobacteria* diversity (Field *et al*. 2015). Combined, this evidence showed that ACIII is not a critical component of the Fe(II) oxidation pathway.

**Figure 8.**
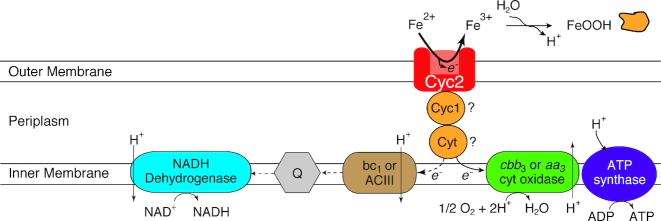
Model for Fe(II) oxidation in the *Zetaproteobacteria* modified from Barco *et al*. (2015). An electron from Fe(II) is passed from Cyc2 to a periplasmic electron carrier (Cyc1 and/or other c-type cytochrome) before being passed to the terminal oxidase (cbb_3_- or aa_3_-type cytochrome c oxidases), generating a proton motive force. For reverse electron transport, the electron from the periplasmic carrier is passed to the bc_1_ complex or alternative complex III (ACIII) before being passed to the quinone pool (Q) where it is used to regenerate NADH.

The putative Fe(II) oxidase, Cyc2, and another cytochrome Cyc1 were first identified in *Zetaproteobacteria* by Barco et al. (2015) through a proteome analysis of PV-1. They were initially identified through comparison of the proteome with the closely related *M. ferrooxydans* M34 genome. Their presence in the proteome suggested that the *cyc1* and *cyc2* genes were missing from the PV-1 draft genome due to gaps in the assembly, which was confirmed by resequencing (Barco *et al*. 2015). The Cyc2 protein from PV-1 is a homolog of the biochemically-characterized Cyc2 Fe(II) oxidase from *Acidithiobacillus ferrooxidans* (22% amino acid identity) (Castelle *et al*. 2008). Based on this, the Fe(II) oxidation pathway model for the *Zetaproteobacteria* was revised (Fig. 8). Cyc2 homologs have been found in other *Zetaproteobacteria* and other neutrophilic FeOB, strengthening the proposed pathway (He *et al*. 2017; Chan *et al*. 2018). In fact, every single ZOTU that has a genomic representative, including ZOTUs without isolates, has a homolog of this putative Fe(II) oxidation gene, consistent with the notion that all *Zetaproteobacteria* are Fe(II)-oxidizers (Field *et al*. 2015; Fullerton *et al*. 2017).

### Genomic clues to niche based on O_2_ and nitrogen

Genomic evidence suggests that adaptation to differing O_2_ conditions plays a role in ZOTU niches. Three terminal oxidases potentially used in the putative Fe oxidation pathway have been found within *Zetaproteobacteria* genomes: cbb_3_- and aa_3_-type cytochrome c oxidases and the cytochrome bd-I ubiquinol oxidase. These have different affinities for oxygen, which would influence the niche of each ZOTU; K_m_’s of 230–300 nM (cbb_3_, bd-I) to 4.3 µM (aa_3_) are reported (Bekker *et al*. 2009; Arai *et al*. 2014). The cbb_3_-type terminal oxidase gene is found in most of the *Zetaproteobacteria*, sometimes in multiple copies, suggesting a predominant preference for very low O_2_ concentrations (submicromolar) (Field *et al*. 2015). However, the complete genomes of *M. aestuarium* and *M. ferrinatatus* contain only the higher-O_2_ adapted aa_3_-type terminal oxidase gene, which helps explain their adaptation to frequently higher O_2_ concentrations of their tidally-mixed water column habitat (Chiu *et al*. 2017). Many *Zetaproteobacteria* genomes have multiple terminal oxidases, suggesting they are adapted to fluctuating oxygen conditions (Field *et al*. 2015; Fullerton *et al*. 2017). ZOTU10 and the isolate *Mariprofundus* sp. DIS-1 may have a higher tolerance for such conditions with increased numbers of genes for O_2_ radical protection (Field *et al*. 2015; Mumford, Adaktylou and Emerson 2016).

The genetic potential for nitrogen species transformations differentiates marine and terrestrial *Zetaproteobacteria*. In the marine environment, most ZOTUs have the potential for assimilatory nitrate reduction to ammonium (*nasA*, *nirBD*) (Field *et al*. 2015; Fullerton *et al*. 2017). In terrestrial Fe(II)-rich springs such as Crystal Geyser, *Zetaproteobacteria* genomes lack these genes, but many possess nitrogen fixation genes (e.g. *nifH*) (Emerson *et al*. 2016; Probst *et al*. 2017). In contrast, only three marine isolates (*Mariprofundus* strains DIS-1, EKF-M39 and M34) and one MAG outside of *Mariprofundus* possesses *nif* genes (Field *et al*. 2015; Mumford, Adaktylou and Emerson 2016; Fullerton *et al*. 2017), and, as yet, it has not been experimentally shown that these isolates fix N_2_. Supporting these genomic observations, the *nifH* gene is rarely detected in marine Fe mats (Jesser *et al*. 2015). From these patterns, the differences between these *Zetaproteobacteria* likely correspond with differences in nitrate and ammonium availability in these habitats; nitrate is below detection at Crystal Geyser compared to concentrations up to 32 µM within Loihi Fe mats (Emerson *et al*. 2016; Sylvan *et al*. 2017). Nitrogen transformations and O_2_ tolerance likely play a role in many *Zetaproteobacteria* niches, though physiological experiments are required for verification. Regardless, there are likely other conditions driving niche diversity yet to be discovered.

## Outstanding questions and opportunities

Over the last two decades, *Zetaproteobacteria* have been established as a diverse, taxonomically-robust class, which thrive in a wide range of Fe(II)-rich habitats. Environmental studies, isolate experiments and genomic analyses have given insight into how they use biomineralization and metabolic strategies to succeed. Building on this work, we are poised to address a number of intriguing questions.

### How did the *Zetaproteobacteria* come to specialize in Fe(II) oxidation?

Thus far, genomic evidence suggests that all *Zetaproteobacteria* are Fe(II)-oxidizers. If this is true, the *Zetaproteobacteria* would be an interesting model system in which to explore the selection and evolution of a particular metabolic specialty. The answer to this question likely rests on the complex challenges of Fe(II) oxidation at circumneutral pH. *Zetaproteobacteria* must position themselves at specific environmental interfaces to gain energy from Fe(II) oxidation. Meanwhile, they must compete with or tolerate abiotic reactions of Fe(II) with O_2_ and nitrogen compounds, which can form O_2_ radicals and toxic nitric and nitrous oxides (Winterbourn 1995; Jones *et al*. 2015). They produce intricate biomineral structures, which allow them to avoid encrustation, control motility and construct mats. Thus, microbial Fe(II) oxidation appears to be a complex physiological trait, which is much more likely to be inherited vertically through descent rather than transmitted horizontally (Martiny, Treseder and Pusch 2013). Since Fe(II) oxidation is a complex trait, this capability was likely acquired by the Zetaproteobacteria prior to their divergence. It is unclear where the Fe(II) oxidation trait originated, but as we determine its genetic basis, phylogenetic comparisons of these genes will allow us to understand FeOB evolutionary relationships.

### What are the drivers of *Zetaproteobacteria* diversification?

The *Zetaproteobacteria* have diversified into at least 59 operational taxonomic units, which we can now track using ZetaHunter (McAllister, Moore and Chan 2018). Given the increasing number of available genomes, the next logical step is to develop a systematic taxonomy based on both 16S rRNA gene and phylogenomics analysis. Ultimately, diversification is driven by the range in environmental niches. We will improve our understanding as we continue to study environmental distribution, physiology of new isolates, and genomes, especially as we focus our explorations beyond the well-studied hydrothermal vents. We may be able to define niches better via discrete sampling, though there are practical lower limits to sample size and spatial resolution. Although intact samples are challenging to obtain, the effort is worthwhile in order to use imaging-based techniques (e.g. FISH), coupled to high spatial-resolution geochemistry and activity measurements (e.g. elemental mapping, SIP) to discern millimeter- and micron-scale associations. We are just beginning to discover the variety of adaptations across genomes. As genome analyses progress, patterns of functional genes and phylogeny will elucidate the drivers of *Zetaproteobacteria* diversification. In turn, genomic clues can help us culture novel organisms, which will be key to demonstrating particular biogeochemical roles. The integrated results of these studies will show how these organisms have evolved to occupy particular niches, and how they could work together to influence the geochemistry of Fe(II)-rich habitats.

### How do *Zetaproteobacteria* affect geochemical cycling, and how can we track these effects?

Now that we know the basics of *Zetaproteobacteria* metabolisms and potential geochemical effects, we can move toward detecting this influence in the environment and determining the controls on those effects. The key will be developing ways to track *Zetaproteobacteria* activity, and relating this to quantitative effects. There is no clear biotic Fe isotopic signature that can be used to assess the activity of microbially mediated Fe(II) oxidation (Anbar 2004). An alternative is to track activity via gene expression. Traditionally, this would be done via a marker gene for Fe(II) oxidation. The *cyc2* gene may work if its expression proves to be specific to Fe(II) oxidation. However, now with (meta)transcriptomic approaches, we can use multiple genes (e.g. the whole Fe(II) oxidation pathway, linked with C fixation and other pathways). With the *Zetaproteobacteria*, this will be an iterative exercise, as we are still determining/validating the genes involved in Fe(II) oxidation and other metabolisms. This will be most straightforward in *Zetaproteobacteria*-dominated hydrothermal Fe mat environments, but work in other environments will improve our understanding of the range of their effects on geochemical cycling. As *Zetaproteobacteria* are widespread in diverse environments, continued work will most likely reveal their broad influence on Fe cycling in marine and saline terrestrial environments.

## Supplementary Material

Supplemental Files
