# Supplementary material for: The Fe(II)-oxidizing Zetaproteobacteria: historical, ecological and genomic perspectives
Source: FEMS Microbiol Ecol. 2019 Jan 30;95(4):fiz015. doi: 10.1093/femsec/fiz015 (PMC6443915; doi:10.1093/femsec/fiz015)
Supplement: Supplemental Files [file fiz015_supplemental_files.zip › SupplementalDocument.docx]

Supplemental Text, Figures, and Table for

**The Fe(II)-oxidizing *Zetaproteobacteria*: Historical, ecological, and genomic perspectives**

Sean M. McAllister,^a^ Ryan M. Moore,^b^ Amy Gartman,^a^* George W. Luther, III,^a^ David Emerson,^c^ and Clara S. Chan^a,d^#

^a^School of Marine Science and Policy, University of Delaware, Newark, Delaware, USA

^b^Center for Bioinformatics and Computational Biology, University of Delaware, Newark, Delaware, USA

^c^Bigelow Laboratory for Ocean Sciences, East Boothbay, Maine, USA

^d^Department of Geological Sciences, University of Delaware, Newark, Delaware,

USA

#Address correspondence to Clara S. Chan, cschan@udel.edu.

*Present address: Amy Gartman, USGS Pacific Coastal and Marine Science Center, Santa Cruz, California, USA

**Document includes:**

- 3 Supplemental Figures
- 1 Supplemental Table
- Supplemental Methods
- Supplemental References

**Supplemental Methods**

***M. ferrooxydans* PV-1 Fe(II) oxidation kinetics**

Prior to each kinetics experiment, fresh PV-1 cells were grown up overnight (approx. 19 hours) on Fe(0) plates following Emerson and Floyd (2005). For each 20 mL electrochemistry cell (consisting of the working, counter, and reference electrodes), between 1 and 13 mL of cell suspension were added to fresh ASW (avg. 4.1•10^4^ cells mL^-1^). Following this, the electrochemistry cell was set up with O_2_ maintained at the desired concentrations using a gas regulator (air:N_2_ gas mix) with continual stirring. At the start of each 1-2 hour experiment, 300 µL of 10.6 mM ferrous ammonium sulfate was added to the electrochemistry cell (final concentration 154 µM Fe(II)). Fe(II) was measured throughout the experiment using cyclic voltammetry following Luther et al. (2008). This experiment represents total abiotic and biotic Fe(II) oxidation. At the end of each run, abiotic oxidation was measured by killing the cells with 1 mM sodium azide for a few min, adding additional ferrous ammonium sulfate, and running the experiment again. At the end of the living (total, biotic + abiotic) and killed runs, cells were fixed in a 1.6% paraformaldehyde solution before being Syto 13-labeled and counted using a Petroff-Hausser counting chamber.

After the kinetics experiments were run, Fe(II) oxidation rates were calculated by determining the pseudo-first-order rate constant (k_1_) from the log-linear slope of the best fit trend-line. These were converted into the rates shown by multiplying by the Fe(II) concentration. The percent of biotic Fe(II) oxidation was calculated by subtracting the abiotic Fe(II) oxidation rate (killed run) from the total rate (living run), and dividing this biotic-only rate by the total rate. These kinetics experiments were run in a similar manner to Druschel et al. (2008) and Rentz et al. (2007).

**Discovering and assessing Zetaproteobacteria in databases**

Full-length Zetaproteobacteria 16S rRNA gene sequences were obtained from the SILVA database (release 128; Glöckner et al., 2017) as well as isolate and single amplified genomes (SAG) from the integrated microbial genomes (IMG) database (Chen et al., 2017). Partial length 16S rRNA gene sequences were obtained primarily from the SILVA database (release 128) as well as sequences pulled from the National Center for Biotechnology Information (NCBI) Sequence Read Archive (SRA) using the Zetaproteobacteria taxonomic query from the Integrated Microbial Next Generation Sequencing (IMNGS) database (version 1.0 build 1710; Lagkouvardos et al., 2016). Only those SRA samples with a significant number of Zetaproteobacteria reads are reported in Dataset S1. All sequences were assigned to ZOTUs using ZetaHunter version 0.3.0, database v.3.

**Phylogenetic trees**

All full-length 16S rRNA gene sequences found in the ZetaHunter database were used in the construction of the phylogenetic tree shown in Figures 5AB and Figure S2, in addition to the outgroups paired with that database. SINA-aligned sequences (Pruesse et al., 2012) were masked using the same 1,282 bp mask used in McAllister et al. (2011). The maximum likelihood phylogenetic tree was constructed using RAxML version 8.2.8, using the general time reversible (GTR) model with CAT approximation and 300 bootstraps (Stamatakis, 2006). The phylogenetic trees in Figures 5AB and Figure S2 are identical, only displayed in different manners (radial vs. rectangular tree layouts). The 16S rRNA gene phylogenetic trees were colored automatically using the Iroki program (Moore et al., 2017).

The concatenated ribosomal tree was constructed using 12 large and small subunit ribosomal proteins (L5p, L6p, L14p, L15p, L17p, L18p, L24p, L30p, S4p, S5p, S11p, and S13p). These ribosomal proteins were chosen because they could be found in most isolate and single amplified genome (SAG) reference genomes, allowing for proper assignment of ZOTU affiliation. Genomes in the tree include all isolates (except GSB-2, which is 48% complete), many SAGs (Field et al., 2015; Scott et al., 2015), and a collection of metagenome assembled genomes (MAGs) from Loihi Seamount (Fullerton et al., 2017), Crystal Geyser (Emerson et al., 2016; Probst et al., 2017; Probst et al., 2018), the Piccard vent field (Anderson et al., 2017), and Painkalac Creek, Australia (Lin et al., 2018). To construct this tree, each ribosomal protein database was aligned individually using MUSCLE (Edgar, 2004), was manually curated, then concatenated into a single alignment file with all 12 ribosomal protein alignments. A phylogenetic tree was then built from this concatenated alignment with RAxML version 7.2.8, using the GTR model with GAMMA approximation and 100 bootstraps (Stamatakis, 2006).

**ZOTU-association networks**

For the ZOTU network in Figure 7, all full and partial length sequences from the SILVA database (release 128) were included (Glöckner *et al.* 2017). For the ZOTU network in Figure S3, only discrete Fe mat samples taken from a small volume (50-75 mL) were included (Fleming *et al.* 2013; Field *et al.* 2015; Scott *et al.* 2015; Fullerton *et al.* 2017; Hager *et al.* 2017; Scott, Glazer and Emerson 2017). Network connections were assigned using ZetaHunter (McAllister, Moore and Chan 2018), which produces edge and node files as an output. ZOTU-association network visualization was done in Cytoscape version 3.4.0. Figure 7 was organized spatially using edge-weighted spring embedded layout, which caused ZOTUs found together in multiple samples to be clustered closer together. Figure S3 was organized spatially using attribute circle layout, which placed ZOTUs with a similar dominant sample type close together.

**Supplemental references**

The following references are listed for all supplemental material (Supplemental Methods, Figures, Table, and Dataset).

Anderson RE, J Reveillaud, E Reddington *et al.* Genomic variation in microbial populations inhabiting the marine subseafloor at deep-sea hydrothermal vents. *Nat Commun* 2017;8:1114. doi:10.1038/s41467-017-01228-6

Baquiran J-PM, Ramírez GA, Haddad AG *et al.* Temperature and redox effect on mineral colonization in Juan de Fuca Ridge flank subsurface crustal fluids. *Front Microbiol* 2016;7:396. doi:10.3389/fmicb.2016.00396

Barco RA, Hoffman CL, Ramírez GA *et al.* *In-situ* incubation of iron-sulfur mineral reveals a diverse chemolithoautotrophic community and a new biogeochemical role for *Thiomicrospira*. *Environ Microbiol* 2017;**19**:1322–37. doi:10.1111/1462-2920.13666

Breier JA, Gomez-Ibanez D, Reddington E *et al.* A precision multi-sampler for deep-sea hydrothermal microbial mat studies. *Deep Sea Res Part I Oceanogr Res Pap* 2012;**70**:83–90. doi:10.1016/j.dsr.2012.10.006

Bowman JP, McCuaig RD. Biodiversity, community structural shifts, and biogeography of prokaryotes within Antarctic continental shelf sediment. *Appl Environ Microbiol* 2003;**69**:2463–83. doi:10.1128/AEM.69.5.2463

Bowman JP, Nowak B. Salmonid gill bacteria and their relationship to amoebic gill disease. *J Fish Dis* 2004;**27**:483–92. doi:10.1111/j.1365-2761.2004.00569.x

Ceh J, van Keulen M, Bourne DG. Coral-associated bacterial communities on Ningaloo Reef, Western Australia. *FEMS Microbiol Ecol* 2011;**75**:134–44. doi:10.1111/j.1574-6941.2010.00986.x

Chang Y-H, Cheng T-W, Lai W-J *et al.* Microbial methane cycling in a terrestrial mud volcano in eastern Taiwan. *Environ Microbiol* 2012;**14**:895–908. doi:10.1111/j.1462-2920.2011.02658.x

Chan CS, Fakra SC, Emerson D *et al.* Lithotrophic iron-oxidizing bacteria produce organic stalks to control mineral growth: implications for biosignature formation. *ISME J* 2011;**5**:717–27. doi:10.1038/ismej.2010.173

Chan CS, McAllister SM, Leavitt AH *et al.* The architecture of iron microbial mats reflects the adaptation of chemolithotrophic iron oxidation in freshwater and marine environments. *Front Microbiol* 2016;**7**:796. doi:10.3389/fmicb.2016.00796

Chen I-MA, Markowitz VM, Chu K *et al.* IMG/M: Integrated genome and metagenome comparative data analysis system. *Nucleic Acids Res* 2017;**45**:D507–16. doi:10.1093/nar/gkw929

Chiu BK, Kato S, McAllister SM *et al.* Novel pelagic iron-oxidizing Zetaproteobacteria from the Chesapeake Bay oxic-anoxic transition zone. *Front Microbiol* 2017;**8**:1280. doi:10.3389/fmicb.2017.01280

Colman DR, Garcia JR, Crossey LJ *et al.* An analysis of geothermal and carbonic springs in the western United States sustained by deep fluid inputs. *Geobiology* 2014;**12**:83–98. doi:10.1111/gbi.12070

Dang H, Li T, Chen M *et al.* Cross-ocean distribution of Rhodobacterales bacteria as primary surface colonizers in temperate coastal marine waters. *Appl Environ Microbiol* 2008;**74**:52–60. doi:10.1128/AEM.01400-07

Dang H, Chen R, Wang L *et al.* Molecular characterization of putative biocorroding microbiota with a novel niche detection of Epsilon- and Zetaproteobacteria in Pacific Ocean coastal seawaters. *Environ Microbiol* 2011;**13**:3059–74. doi:10.1111/j.1462-2920.2011.02583.x

Davis RE, Moyer CL. Extreme spatial and temporal variability of hydrothermal microbial mat communities along the Mariana Island Arc and southern Mariana back-arc system. *J Geophys Res* 2008;**113**:B08S15. doi:10.1029/2007JB005413

Davis RE, Stakes DS, Wheat CG *et al.* Bacterial variability within an iron-silica-manganese-rich hydrothermal mound located off-axis at the Cleft segment, Juan de Fuca Ridge. *Geomicrobiol J* 2009;**26**:570–80. doi:10.1080/01490450902889080

Dekas AE, Connon SA, Chadwick GL *et al.* Activity and interactions of methane seep microorganisms assessed by parallel transcription and FISH-NanoSIMS analyses. *ISME J* 2016;**10**:678–92. doi:10.1038/ismej.2015.145

Dhillon A, Teske A, Dillon J *et al.* Molecular characterization of sulfate-reducing bacteria in the Guaymas Basin. *Appl Environ Microbiol* 2003;**69**:2765–72. doi:10.1128/AEM.69.5.2765

Diaby N, Dold B, Rohrbach E *et al.* Temporal evolution of bacterial communities associated with the *in situ* wetland-based remediation of a marine shore porphyry copper tailings deposit. *Sci Total Environ* 2015;**533**:110–21. doi:10.1016/j.scitotenv.2015.06.076

Druschel GK, Emerson D, Sutka R *et al.* Low-oxygen and chemical kinetic constraints on the geochemical niche of neutrophilic iron(II) oxidizing microorganisms. *Geochim Cosmochim Acta* 2008;**72**:3358–70.

Durand L, Zbinden M, Cueff-Gauchard V *et al.* Microbial diversity associated with the hydrothermal shrimp *Rimicaris exoculata* gut and occurrence of a resident microbial community. *FEMS Microbiol Ecol* 2010;**71**:291–303. doi:10.1111/j.1574-6941.2009.00806.x

Durand L, Roumagnac M, Cueff-Gauchard V *et al.* Biogeographical distribution of Rimicaris exoculata resident gut epibiont communities along the Mid-Atlantic Ridge hydrothermal vent sites. *FEMS Microbiol Ecol* 2015;**91**:fiv101. doi:10.1093/femsec/fiv101

Eder W, Jahnke LL, Schmidt M *et al.* Microbial diversity of the brine-seawater interface of the Kebrit Deep, Red Sea, studied via 16S rRNA gene sequences and cultivation methods. *Appl Environ Microbiol* 2001;**67**:3077–85. doi:10.1128/AEM.67.7.3077

Edgar RC. MUSCLE: multiple sequence alignment with high accuracy and high throughput. *Nucleic Acids Res* 2004:32:1792–7. doi:10.1093/nar/gkh340

Edwards KJ, Glazer BT, Rouxel OJ *et al.* Ultra-diffuse hydrothermal venting supports Fe-oxidizing bacteria and massive umber deposition at 5000 m off Hawaii. *ISME J* 2011;**5**:1748–58. doi:10.1038/ismej.2011.48

Emerson D, Floyd MM. Enrichment and isolation of iron-oxidizing bacteria at neutral pH. *Methods Enzymol* 2005;**397**:112–23. doi:10.1016/S0076-6879(05)97006-7

Emerson D, Moyer CL. Neutrophilic Fe-oxidizing bacteria are abundant at the Loihi Seamount hydrothermal vents and play a major role in Fe oxide deposition. *Appl Environ Microbiol* 2002;**68**:3085–93. doi:10.1128/AEM.68.6.3085

Emerson D, Rentz JA, Lilburn TG *et al.* A novel lineage of proteobacteria involved in formation of marine Fe-oxidizing microbial mat communities. *PLoS One* 2007;**2**:e667. doi:10.1371/journal.pone.0000667

Emerson JB, Thomas BC, Alvarez W *et al.* Metagenomic analysis of a high carbon dioxide subsurface microbial community populated by chemolithoautotrophs and bacteria and archaea from candidate phyla. *Environ Microbiol* 2016;**18**:1686–703. doi:10.1111/1462-2920.12817

Field EK, Sczyrba A, Lyman AE *et al.* Genomic insights into the uncultivated marine Zetaproteobacteria at Loihi Seamount. *ISME J* 2015;**9**:857–70. doi:10.1038/ismej.2014.183

Fleming EJ, Davis RE, McAllister SM *et al.* Hidden in plain sight: discovery of sheath-forming, iron-oxidizing Zetaproteobacteria at Loihi Seamount, Hawaii, USA. *FEMS Microbiol Ecol* 2013;**85**:116–27. doi:10.1111/1574-6941.12104

Flores GE, Campbell JH, Kirshtein JD *et al.* Microbial community structure of hydrothermal deposits from geochemically different vent fields along the Mid-Atlantic Ridge. *Environ Microbiol* 2011;**13**:2158–71. doi:10.1111/j.1462-2920.2011.02463.x

Forget NL, Murdock SA, Juniper SK. Bacterial diversity in Fe-rich hydrothermal sediments at two South Tonga Arc submarine volcanoes. *Geobiology* 2010;**8**:417–32. doi:10.1111/j.1472-4669.2010.00247.x

Freeman SA, Sierra-Alvarez R, Altinbas M *et al.* Molecular characterization of mesophilic and thermophilic sulfate reducing microbial communities in expanded granular sludge bed (EGSB) reactors. *Biodegradation* 2008;**19**:161–77. doi:10.1007/s10532-007-9123-9

Fullerton H, Hager KW, McAllister SM *et al.* Hidden diversity revealed by genome-resolved metagenomics of iron-oxidizing microbial mats from Lō’ihi Seamount, Hawai’i. *ISME J* 2017;**11**:1900–14. doi:10.1038/ismej.2017.40

Gillan DC, Speksnijder AGCL, Zwart G *et al.* Genetic diversity of the biofilm covering Montacuta ferruginosa (Mollusca, Bivalvia) as evaluated by denaturing gradient gel electrophoresis analysis and cloning of PCR-amplified gene fragments coding for 16S rRNA. *Appl Environ Microbiol* 1998;**64**:3464–72.

Glöckner FO, Yilmaz P, Quast C *et al.* 25 years of serving the community with ribosomal RNA gene reference databases and tools. *J Biotechnol* 2017;**261**:169–76. doi:10.1016/j.jbiotec.2017.06.1198

Gonnella G, Böhnke S, Indenbirken D *et al.* Endemic hydrothermal vent species identified in the open ocean seed bank. *Nat Microbiol* 2016;**1**:16086. doi:10.1038/nmicrobiol.2016.86

Guan Y, T Hikmawan, A Antunes *et al.* Diversity of methanogens and sulfate-reducing bacteria in the interfaces of five deep-sea anoxic brines of the Red Sea. *Res Microbiol* 2015;166:688–99. doi:10.1016/j.resmic.2015.07.002

Hager KW, Fullerton H, Butterfield DA *et al.* Community structure of lithotrophically-driven hydrothermal microbial mats from the Mariana Arc and Back-Arc. *Front Microbiol* 2017;**8**:1578. doi:10.3389/fmicb.2017.01578

Handley KM, Boothman C, Mills RA *et al.* Functional diversity of bacteria in a ferruginous hydrothermal sediment. *ISME J* 2010;**4**:1193–205. doi:10.1038/ismej.2010.38

Harrison BK, Orphan VJ. Method for assessing mineral composition- dependent patterns in microbial diversity using magnetic and density separation. *Geomicrobiol J* 2012;**29**:435–49. doi:10.1080/01490451.2011.581327

Hassenrück C, Fink A, Lichtschlag A *et al.* Quantification of the effects of ocean acidification on sediment microbial communities in the environment: The importance of ecosystem approaches. *FEMS Microbiol Ecol* 2016;**92**:fiw02. doi:10.1093/femsec/fiw027

Heim C, Simon K, Ionescu D *et al.* Assessing the utility of trace and rare earth elements as biosignatures in microbial iron oxyhydroxides. *Front Earth Sci* 2015;**3**:6. doi:10.3389/feart.2015.00006

Henri PA, Rommevaux-Jestin C, Lesongeur F *et al.* Structural iron (II) of basaltic glass as an energy source for Zetaproteobacteria in an abyssal plain environment, off the Mid Atlantic Ridge. *Front Microbiol* 2016;**6**:1518. doi:10.3389/fmicb.2015.01518

Hodges TW, Olson JB. Molecular comparison of bacterial communities within iron-containing flocculent mats associated with submarine volcanoes along the Kermadec Arc. *Appl Environ Microbiol* 2009;**75**:1650–7. doi:10.1128/AEM.01835-08

Hoshino T, Kuratomi T, Morono Y *et al.* Ecophysiology of Zetaproteobacteria associated with shallow hydrothermal iron-oxyhydroxide deposits in Nagahama Bay of Satsuma Iwo-Jima, Japan. *Front Microbiol* 2016;**6**:1554. doi:10.3389/fmicb.2015.01554

Huber JA, Morrison HG, Huse SM *et al.* Effect of PCR amplicon size on assessments of clone library microbial diversity and community structure. *Environ Microbiol* 2009;**11**:1292–302. doi:10.1111/j.1462-2920.2008.01857.x

Ionescu D, Heim C, Polerecky L *et al.* Diversity of iron oxidizing and reducing bacteria in flow reactors in the Äspö Hard Rock Laboratory. *Geomicrobiol J* 2015;**32**:207–20. doi:10.1080/01490451.2014.884196

Jägevall S, Rabe L, Pedersen K. Abundance and diversity of biofilms in natural and artificial aquifers of the Äspö Hard Rock Laboratory, Sweden. *Microb Ecol* 2011;**61**:410–22. doi:10.1007/s00248-010-9761-z

Jungbluth SP, Lin H-T, Cowen JP *et al.* Phylogenetic diversity of microorganisms in subseafloor crustal fluids from Holes 1025C and 1026B along the Juan de Fuca Ridge flank. *Front Microbiol* 2014;**5**:119. doi:10.3389/fmicb.2014.00119

Kato S, Yanagawa K, Sunamura M *et al.* Abundance of Zetaproteobacteria within crustal fluids in back-arc hydrothermal fields of the Southern Mariana Trough. *Environ Microbiol* 2009a;**11**:3210–22. doi:10.1111/j.1462-2920.2009.02031.x

Kato S, Kobayashi C, Kakegawa T *et al.* Microbial communities in iron-silica-rich microbial mats at deep-sea hydrothermal fields of the Southern Mariana Trough. *Environ Microbiol* 2009b;**11**:2094–111. doi:10.1111/j.1462-2920.2009.01930.x

Kato S, Ikehata K, Shibuya T *et al.* Potential for biogeochemical cycling of sulfur, iron and carbon within massive sulfide deposits below the seafloor. *Environ Microbiol* 2015;**17**:1817–35. doi:10.1111/1462-2920.12648

Kawagucci S, Yoshida YT, Noguchi T *et al.* Disturbance of deep-sea environments induced by the M9.0 Tohoku Earthquake. *Sci Rep* 2012;**2**:270. doi:10.1038/srep00270

Kawahara N, Shigematsu K, Miyadai T *et al.* Comparison of bacterial communities in fish farm sediments along an organic enrichment gradient. *Aquaculture* 2009;**287**:107–13. doi:10.1016/j.aquaculture.2008.10.003

Koren O, Rosenberg E. Bacteria associated with the bleached and cave coral Oculina patagonica. *Microb Ecol* 2008;**55**:523–9. doi:10.1007/s00248-007-9297-z

Kormas KA, Tamaki H, Hanada S *et al.* Apparent richness and community composition of Bacteria and Archaea in geothermal springs. *Aquat Microb Ecol* 2009;**57**:113–22. doi:10.3354/ame01333

Koyama S, Konishi M, Ohta Y *et al.* Attachment and detachment of living microorganisms using a potential-controlled electrode. *Mar Biotechnol* 2013;**15**:461–75. doi:10.1007/s10126-013-9495-2

Kumar N, Omoregie EO, Rose J *et al.* Inhibition of sulfate reducing bacteria in aquifer sediment by iron nanoparticles. *Water Res* 2014;**51**:64–72. doi:10.1016/j.watres.2013.09.042

Lagkouvardos I, Joseph D, Kapfhammer M *et al.* IMNGS: A comprehensive open resource of processed 16S rRNA microbial profiles for ecology and diversity studies. *Sci Rep* 2016;**6**:33721. doi:10.1038/srep33721

Lambais MR, Otero XL, Cury JC. Bacterial communities and biogeochemical transformations of iron and sulfur in a high saltmarsh soil profile. *Soil Biol Biochem* 2008;**40**:2854–64. doi:10.1016/j.soilbio.2008.08.014

Laufer K, Nordhoff M, Halama M *et al.* Microaerophilic Fe(II)-oxidizing Zetaproteobacteria isolated from low-Fe marine coastal sediments: Physiology and characterization of their twisted stalks. *Appl Environ Microbiol* 2017;**83**:e03118-16. doi:10.1128/AEM.03118-16

Li J, Zhou H, Peng X *et al.* Microbial diversity and biomineralization in low-temperature hydrothermal iron-silica-rich precipitates of the Lau Basin hydrothermal field. *FEMS Microbiol Ecol* 2012;**81**:205–16. doi:10.1111/j.1574-6941.2012.01367.x

Li J, Peng X, Zhou H *et al.* Molecular evidence for microorganisms participating in Fe, Mn, and S biogeochemical cycling in two low-temperature hydrothermal fields at the Southwest Indian Ridge. *J Geophys Res Biogeosciences* 2013;**118**:665–79. doi:10.1002/jgrg.20057

Lin W, W Zhang, X Zhao *et al.* Genomic expansion of magnetotactic bacteria reveals an early common origin of magnetotaxis with lineage-specific evolution. *ISME J* 2018;12:1508–19. doi:10.1038/s41396-018-0098-9

Lokmer A, Wegner KM. Hemolymph microbiome of Pacific oysters in response to temperature, temperature stress and infection. *ISME J* 2015;**9**:670–82. doi:10.1038/ismej.2014.160

Luther, III GW, Rickard DT, Theberge S *et al.* Determination of metal (bi)sulfide stability constants of Mn^2+^, Fe^2+^, Co^2+^, Ni^2+^, Cu^2+^, and Zn^2+^ by voltammetric methods. *Environ Sci Technol* 1996;**30**:671–9. doi:10.1021/es950417i

Luther, III GW, Glazer BT, Ma S *et al*. Use of voltammetric solid-state (micro)electrodes for studying biogeochemical processes: Laboratory measurements to real time measurements with an *in situ* electrochemical analyzer (ISEA). *Mar Chem* 2008;**108**:221–35.

Makita H, Kikuchi S, Mitsunobu S *et al.* Comparative analysis of microbial communities in iron-dominated flocculent mats in deep-sea hydrothermal environments. *Appl Environ Microbiol* 2016;**82**:5741–55. doi:10.1128/AEM.01151-16

Makita H, Tanaka E, Mitsunobu S *et al.* *Mariprofundus micogutta* sp. nov., a novel iron-oxidizing zetaproteobacterium isolated from a deep-sea hydrothermal field at the Bayonnaise knoll of the Izu-Ogasawara arc, and a description of Mariprofundales ord. nov. and Zetaproteobacteria classis. *Arch Microbiol* 2017;**199**:335–46. doi:10.1007/s00203-016-1307-4

Marty F, Gueuné H, Malard E *et al.* Identification of key factors in accelerated low water corrosion through experimental simulation of tidal conditions: Influence of stimulated indigenous microbiota. *Biofouling* 2014;**30**:281–97. doi:10.1080/08927014.2013.864758

McAllister SM, Davis RE, McBeth JM *et al.* Biodiversity and emerging biogeography of the neutrophilic iron-oxidizing Zetaproteobacteria. *Appl Environ Microbiol* 2011;**77**:5445–57. doi:10.1128/AEM.00533-11

McAllister SM, Barnett JM, Heiss JW *et al.* Dynamic hydrologic and biogeochemical processes drive microbially enhanced iron and sulfur cycling within the intertidal mixing zone of a beach aquifer. *Limnol Oceanogr* 2015;**60**:329–45. doi:10.1111/lno.10029

McAllister SM, Moore RM, and Chan CS. ZetaHunter, a reproducible taxonomic classification tool for tracking the ecology of the Zetaproteobacteria and other poorly resolved taxa. *Microbiol Res Announc* 2018;**7**:e00932-18. doi:10.1128/MRA.00932-18

McBeth JM, Little BJ, Ray RI *et al.* Neutrophilic iron-oxidizing “Zetaproteobacteria” and mild steel corrosion in nearshore marine environments. *Appl Environ Microbiol* 2011;**77**:1405–12. doi:10.1128/AEM.02095-10

Meyer-Dombard DR, Amend JP, Osburn MR. Microbial diversity and potential for arsenic and iron biogeochemical cycling at an arsenic rich, shallow-sea hydrothermal vent (Tutum Bay, Papua New Guinea). *Chem Geol* 2013;**348**:37–47. doi:10.1016/j.chemgeo.2012.02.024

Moore RM, Harrison AO, McAllister SM *et al.* Iroki: automatic customization for phylogenetic trees. *bioRxiv* 2017, doi:10.1101/106138

Mori JF, Scott JJ, Hager KW *et al.* Physiological and ecological implications of an iron- or hydrogen-oxidizing member of the Zetaproteobacteria, *Ghiorsea bivora*, gen. nov., sp. nov. *ISME J* 2017;**11**:2624–36. doi:10.1038/ismej.2017.132

Moyer CL, Dobbs FC, Karl DM. Phylogenetic diversity of the bacterial community from a microbial mat at an active, hydrothermal vent system, Loihi Seamount, Hawaii. *Appl Environ Microbiol* 1995;**61**:1555–62.

Mumford AC, Adaktylou IJ, Emerson D. Peeking under the iron curtain: development of a microcosm for imaging the colonization of steel surfaces by *Mariprofundus* sp. strain DIS-1, an oxygen-tolerant Fe-oxidizing bacterium. *Appl Environ Microbiol* 2016;**82**:6799–807. doi:10.1128/AEM.01990-16

Ogawa M, Tanimoto D, Okamoto T *et al.* Ester-linked phospholipid fatty acids in marine sediments: Testing the possibility of methane seepage in the Japan Sea. *Aquat Ecosyst Health Manag* 2003;**6**:397–408. doi:10.1080/714044169

Omoregie EO, Mastalerz V, de Lange G *et al.* Biogeochemistry and community composition of iron- and sulfur-precipitating microbial mats at the Chefren mud volcano (Nile deep sea fan, eastern Mediterranean). *Appl Environ Microbiol* 2008;**74**:3198–215. doi:10.1128/AEM.01751-07

Pedersen K, Arlinger J, Ekendahl S *et al.* 16S rRNA gene diversity of attached and unattached bacteria in boreholes along the access tunnel to the Äspö hard rock laboratory, Sweden. *FEMS Microbiol Ecol* 1996;**19**:249–62. doi:10.1111/j.1574-6941.1996.tb00217.x

Pischedda L, Militon C, Gilbert F *et al.* Characterization of specificity of bacterial community structure within the burrow environment of the marine polychaete Hediste (Nereis) diversicolor. *Res Microbiol* 2011;**162**:1033–42. doi:10.1016/j.resmic.2011.07.008

Probst AJ, Castelle CJ, Singh A *et al.* Genomic resolution of a cold subsurface aquifer community provides metabolic insights for novel microbes adapted to high CO_2_ concentrations. *Environ Microbiol* 2017;**19**:459–74. doi:10.1111/1462-2920.13362

Probst AJ, B Ladd, JK Jarett *et al.* Differential depth distribution of microbial function and putative symbionts through sediment-hosted aquifers in the deep terrestrial subsurface. *Nat Microbiol* 2018;3:328–36. doi:10.1038/s41564-017-0098-y

Pruesse E, Peplies J, Glöckner FO. SINA: Accurate high-throughput multiple sequence alignment of ribosomal RNA genes. *Bioinformatics* 2012;**28**:1823–9. doi:10.1093/bioinformatics/bts252

Ramos-Padrón E, Bordenave S, Lin S *et al.* Carbon and sulfur cycling by microbial communities in a gypsum-treated oil sands tailings pond. *Env Sci Technol* 2011;**45**:439–46. doi:10.1021/es1028487

Rassa AC, McAllister SM, Safran SA *et al.* Zeta-Proteobacteria dominate the colonization and formation of microbial mats in low-temperature hydrothermal vents at Loihi Seamount, Hawaii. *Geomicrobiol J* 2009;**26**:623–38. doi:10.1080/01490450903263350

Reis MP, Dias MF, Costa PS *et al.* Metagenomic signatures of a tropical mining-impacted stream reveal complex microbial and metabolic networks. *Chemosphere* 2016;**161**:266–73. doi:10.1016/j.chemosphere.2016.06.097

Rentz JA, Kraiya C, Luther GW III, and Emerson D. Control of ferrous iron oxidation within circumneutral microbial iron mats by cellular activity and autocatalysis. *Environ Sci Technol* 2007;**41**:6084–89.

Vander Roost J, Thorseth IH, Dahle H. Microbial analysis of Zetaproteobacteria and co-colonizers of iron mats in the Troll Wall Vent Field, Arctic Mid-Ocean Ridge. *PLoS One* 2017;**12**:e0185008. doi:10.1371/journal. pone.0185008

Rubin-Blum M, Antler G, Tsadok R *et al.* First evidence for the presence of iron oxidizing Zetaproteobacteria at the Levantine continental margins. *PLoS One* 2014;**9**:e91456. doi:10.1371/journal.pone.0091456

Ruff SE, Arnds J, Knittel K *et al.* Microbial communities of deep-sea methane seeps at Hikurangi continental margin (New Zealand). *PLoS One* 2013;**8**:e72627. doi:10.1371/journal.pone.0072627

Sanguin H, Sarniguet A, Gazengel K *et al.* Rhizosphere bacterial communities associated with disease suppressiveness stages of take-all decline in wheat monoculture. *New Phytol* 2009;**184**:694–707. doi:10.1111/j.1469-8137.2009.03010.x

Santelli CM, Orcutt BN, Banning E *et al.* Abundance and diversity of microbial life in ocean crust. *Nature* 2008;**453**:653–6. doi:10.1038/nature06899

Schindelin J, Arganda-Carreras I, Frise E *et al.* Fiji: an open-source platform for biological-image analysis. *Nat Methods* 2012;**9**:676–82. doi:10.1038/nmeth.2019

Scott JJ, Breier JA, Luther, III GW *et al.* Microbial iron mats at the Mid-Atlantic Ridge and evidence that Zetaproteobacteria may be restricted to iron-oxidizing marine systems. *PLoS One* 2015;**10**:e0119284. doi:10.1371/journal.pone.0119284

Scott JJ, Glazer BT, Emerson D. Bringing microbial diversity into focus: high-resolution analysis of iron mats from the Lō’ihi Seamount. *Environ Microbiol* 2017;**19**:301–16. doi:10.1111/1462-2920.13607

Singer E, Heidelberg JF, Dhillon A *et al.* Metagenomic insights into the dominant Fe(II) oxidizing Zetaproteobacteria from an iron mat at Lō’ihi, Hawai’i. *Front Microbiol* 2013;**4**:52. doi:10.3389/fmicb.2013.00052

Stamatakis A. RAxML-VI-HPC: maximum likelihood-based phylogenetic analyses with thousands of taxa and mixed models. *Bioinformatics* 2006;**22**:2688–90. doi:10.1093/bioinformatics/btl446

Stauffert M, Cravo-Laureau C, Jézéquel R *et al.* Impact of oil on bacterial community structure in bioturbated sediments. *PLoS One* 2013;**8**:e65347. doi:10.1371/journal.pone.0065347

Sudek LA, Templeton AS, Tebo BM *et al.* Microbial ecology of Fe (hydr)oxide mats and basaltic rock from Vailulu’u Seamount, American Samoa. *Geomicrobiol J* 2009;**26**:581–96. doi:10.1080/01490450903263400

Sylvan JB, Toner BM, Edwards KJ. Life and death of deep-sea vents: Bacterial diversity and ecosystem succession on inactive hydrothermal sulfides. *MBio* 2012a;**3**:e00279-11. doi:10.1128/mBio.00279-11

Sylvan JB, Pyenson BC, Rouxel O *et al.* Time-series analysis of two hydrothermal plumes at 9°50’N East Pacific Rise reveals distinct, heterogeneous bacterial populations. *Geobiology* 2012b;**10**:178–92. doi:10.1111/j.1472-4669.2011.00315.x

Taketani RG, Franco NO, Rosado AS *et al.* Microbial community response to a simulated hydrocarbon spill in mangrove sediments. *J Microbiol* 2010;**48**:7–15. doi:10.1007/s12275-009-0147-1

Toner BM, Lesniewski RA, Marlow JJ *et al.* Mineralogy drives bacterial biogeography of hydrothermally inactive seafloor sulfide deposits. *Geomicrobiol J* 2013;**30**:313–26. doi:10.1080/01490451.2012.688925

Unno T, Kim J, Kim Y *et al.* Influence of seawater intrusion on microbial communities in groundwater. *Sci Total Environ* 2015;**532**:337–43. doi:10.1016/j.scitotenv.2015.05.111

Vasquez-Cardenas D, van de Vossenberg J, Polerecky L *et al.* Microbial carbon metabolism associated with electrogenic sulphur oxidation in coastal sediments. *ISME J* 2015;**9**:1966–78. doi:10.1038/ismej.2015.10

Winder RS, Lamarche J, Constabel CP *et al.* The effects of high-tannin leaf litter from transgenic poplars on microbial communities in microcosm soils. *Front Microbiol* 2013;**4**:290. doi:10.3389/fmicb.2013.00290

Yanagawa K, Nunoura T, McAllister SM *et al.* The first microbiological contamination assessment by deep-sea drilling and coring by the D/V Chikyu at the Iheya North hydrothermal field in the Mid-Okinawa Trough (IODP Expedition 331). *Front Microbiol* 2013;**4**:327. doi:10.3389/fmicb.2013.00327

Yanagawa K, Breuker A, Schippers A *et al.* Microbial community stratification controlled by the subseafloor fluid flow and geothermal gradient at the Iheya North hydrothermal field in the Mid-Okinawa Trough (IODP Expedition 331). *Appl Environ Microbiol* 2014;**80**:6126–35. doi:10.1128/AEM.01741-14

Yang C-W, Tang S-L, Chen L-Y *et al.* Removal of nonylphenol by earthworms and bacterial community change. *Int Biodeterior Biodegradation* 2014;**96**:9–17. doi:10.1016/j.ibiod.2014.09.010

Zbinden M, Cambon-Bonavita M-A. Occurrence of Deferribacterales and Entomoplasmatales in the deep-sea Alvinocarid shrimp Rimicaris exoculata gut. *FEMS Microbiol Ecol* 2003;**46**:23–30. doi:10.1016/S0168-6496(03)00176-4
